# Causes of infant deaths and patterns of associated factors in Eastern Ethiopia: Results of verbal autopsy (InterVA-4) study

**DOI:** 10.1371/journal.pone.0270245

**Published:** 2022-08-04

**Authors:** Samuel Mebrahtom, Alemayehu Worku, Daniel J. Gage

**Affiliations:** 1 Ethiopian Institute of Water Resources, Addis Ababa University, Addis Ababa, Ethiopia; 2 School of Public Health, College of Health Sciences, Addis Ababa University, Addis Ababa, Ethiopia; 3 Department of Molecular and Cell Biology, University of Connecticut, Storrs, Connecticut, United States of America; Institute for Health Metrics and Evaluation, UNITED STATES

## Abstract

**Background:**

In a range of setting, detecting and generate empirical information on the cause of infant death and contributing risk factors at population level is basically utmost essential to take evidence-based measures in reducing infant morbidity and mortality. An electronic verbal autopsy is suitable tool and best alternative solution to determine individuals’ cause of death in a setting where the majority of deaths occur at home and civil registration systems do not exist. The present study was undertaken to find out cause of infant death, applying computer-based probabilistic model (InterVA-4) and analyze the patterns of association factors of mother’s and the deceased infant’s characteristics to the leading cause-specific infant mortality in Eastern Ethiopia.

**Methods:**

The study employed a community-based prospective longitudinal survey, which was conducted with routinely enumeration of reported infant deaths for a period of two years (from September 2016 to August 2018) in Eastern part of Ethiopia. Using the two-stage cluster sampling technique, the study was undertaken in four randomly selected districts of West Hararghe zone and two districts of zone 3 in Oromia and Afar regional state, respectively. The study included a total of 362 infants who were deceased during the study period. Data was collected by trained enumerators by interviewing the mothers or guardians of the deceased infant using a 2014 standardize World Health Organization (WHO) Verbal Autopsy questionnaire. InterVA-4 model were used for processing and interpreting verbal autopsy data in order to arrive at the most likely causes of infant death. SPSS version 23 was also used for statistical analysis of frequency distribution and logistic regression for the association between covariates and outcomes.

**Findings:**

Of the overall (362) deceased infants’ during the study period, 53.0% of deaths occurred during neonatal time while 47.0% died in the post-neonatal period. Acute respiratory infection including neonatal and post-neonatal pneumonia (38.4%), birth asphyxia (16.4%), diarrheal diseases (16.3%), prematurity (7.4%) and malaria (4.3%) were found to be the leading causes of infant mortality in the study area. The independent factors strongly associated with probable ARI, including pneumonia related mortality as compared to all-causes of death were infants with maternal age lower than 20 years old (p = 0.001, AOR: 4.82, 95% CI: 1.88, 12.3) and infant being died outside of heath facilities (P = 0.007, AOR: 2.85, 95% CI: 1.33, 6.12). The post-neonatal period (P = 0.000, AOR: 15.5, 95% CI: 6.35, 37.8) and infant died in the wet season (P = 0.006, AOR: 2.38, 95% CI: 1.28, 4.44) had strong relationship with dying from diarrhea-related death than those infants died from all non-diarrhea. The death due to malaria robustly associated with infants whose mothers age between 20–35 years old (P = 0.024, AOR: 4.44, 95% CI: 1.22, 16.2) and infant who was dwelled in the districts of Afar region (P = 0.013, AOR: 4.08, 95% CI: 1.35, 12.4).

**Conclusion:**

The highest cause of infant mortality was associated with disease of respiratory system, particularly acute respiratory infection, including both neonates and post-neonatal pneumonia. Most of the infant deaths existed are as a result of diseases and conditions that are readily preventable or treatable cause, similar to those reported in worldwide, which have needs of further attention. The patterns of significant associated factors across cause-specific mortality against all-cause of death were dissimilar. Therefore, strengthen maternal and child health program with effective preventive interventions emphasizing on the most common cause of infant deaths and those factors contributing in raising mortality risk are required.

## Background

Infant mortality refers to “the death of a live-born infant before their first birthday” [1, 2]. This mortality rate reflect as not only a measure of the risk of infant death but it is used more broadly as a crude indicator of Community health status, Poverty and socioeconomic status levels in a community, Availability and quality of health services and medical technology [3]. It is, therefore, recognized as a uniquely vulnerable to several factors that impact health, represents a long-standing concern of public health and an important marker of the overall health of a society [2].

Globally, around 44% of all under-five deaths occurred during the neonatal period [4] and 75% occurred within the first year of life–with an estimated 4.1 million infants die in 2017 [5]. More than 98% of these deaths occur in developing countries [6], and roughly half of those deaths occurred in sub-Saharan Africa [6, 7]. In worldwide, infant mortality rate has shown decline from the rate of 63 deaths per 1000 live births in 1990 to 29 deaths per 1000 live births in 2017 [5], yet the rate is at highest level in developing world [5, 6].

Evidence showed that Ethiopia has shown a remarkable improvement in the reduction of infant mortality [8–10]. Despite this improvement, however, the death rate of infant is still at the highest level which stands at (59 deaths per 1000 live births) with disproportionately higher amongst population of some regions [9]. Afar and Oromia regions are among the highest in infant mortality. Information on cause of Infant mortality pattern is very much limited, and the available studies were mainly conducted in the hospitals and demographic and health surveillance sites, which make difficulties to understand the cause of death situation in local settings. If these conditions remain as such, proper measures could not be taken and ultimately more Ethiopian children will die. Attention to this condition is important if the Sustainable Development Goal (SDG)-goal 3 target 3.2 (to reduce childhood mortality) is ever to be achieved by 2030 [11].

Infant deaths are intrinsically linked to several causes and factors which need extensive studies. Without addressing this, it’s going to be hard to see the same level of progress that has been made in the reduction of infant mortality in last decade. Cause leads directly to a death, while a contributor is a risk factor that makes the death more likely to occur [12]. Cause of infant death information are critical for formulating good public health policy [13]. Emphatically, detecting and generating empirical information on cause of infant death and patterns of association factors on cause-specific mortality at population level is the first priority and basically utmost essential to take evidence-based measures. This ultimately contributes in reducing infant morbidity and mortality. However, determination of causes of death is a global concern as around half of the world’s children deaths pass without any formal registration of the cause of death [14]. Such situation leads to a major problem for understanding the infant’s health problems in general and difficult for planning the best solutions.

Developing countries have been the worst and serious concern in determining the cause of infant death, and yet little is known about cause of death in sub-Saharan Africa [15]. Ethiopia is one of a nation with lack of consistent cause of death information [16, 17] and understanding the cause of infant death in any setting is still challenging. This is for the reason that most people die at home [16, 18–20] and lost their life without having had contact with the health system [19, 21]. The civil registration systems in general do not exist and there is limited in medical capacity to produce death certificates for the population [16]. As an option, Verbal autopsy is the only available tool and best solution to determine cause of death in a setting where the majority of deaths occur at home and civil registration systems incomplete [13, 22]. For many years, researchers and policy-makers have been undertaking Verbal Autopsy interviews to ascertain the cause of death [23]. But there remains a problem on how to interpret VA interviews reliably and consistently in order to arrive at cause of death. For this effect, InterVA is a suite of computer models for interpreting verbal autopsy data, which recognize a range of indicators relating to a particular death, processes them in a mathematical model based on Bayes’ theorem, and produces as its output likely cause(s) of death [24]. Taking its limitation into account from a validation studies, the 2014 WHO VA instrument is developed to ascertain all individuals cause of death and describes cause-specific mortality fractions at population level [25].

Investigating cause of infant mortality at population level and analyzing the factors contributing to the major cause-specific mortality risk is crucial. There are limited studies on detecting cause of infant mortality at population level and many studies established the risk of dying factors data among those who did not die. No study have been conducted in any sort of settings on the patterns of maternal and infant characteristical factors associated with the most common cause-specific infant deaths against all other cause of death. The objective of the present study, therefore, was to determine causes of infant death using InterVA-4 model and investigate the patterns of maternal and infant characteristics factors on the major cause-specific mortality as compared to mortality from all-causes in Eastern Ethiopia. This study contributes to be filling the knowledge gaps that exist on the pattern of the associated factors among the most common cause-specific infant death against deaths from all other causes. This uniquely would help to better understand the factors that largely have an effect on multiplying infant’s risk of death from specific cause of illness or conditions. This could be important for developing effective and efficient public health strategies and interventions, which will ultimately be reduce the burden of infant mortality.

## Materials and methods

### Study design

The study employed a community-based prospective longitudinal survey, which was conducted with routinely enumeration of reported and notified infant deaths for two years (from September 2016 to August 2018) in Eastern Ethiopia.

### Study area

The study area was geographically located in West Hararghe administrative zone of Oromia region and Zone 3 of Afar region in Eastern part of Ethiopia. This study was conducted in six randomly selected districts; namely Chiro, Mieso, Gemechis and Tullo districts of West Hararghe administrative zone of Oromia as well as Amibara and Awash Fentale districts that comprised in zone 3 administration of Afar region. This study was covering an area of 5561.77km^2^ and 3053.46km^2^ in Oromia and Afar region, respectively. In accordance with the 2007 National Population and Housing Census of Ethiopia [26], the total projected population estimate for 2016 in the study area of oromia is 836,107 while 118,423 in Afar. Of the total population, 3.4% are under the age of one year in the national context and more than 90 percent of the populations are rural dwellers [26].

The populations in the districts are characterized by a diversified of economic activities such as cultivation of food crops (mainly, maize and sorghum) and cash crops (coffee and “chat”), mixed livestock, labor and trading. The study area has two main climate seasons—a dry season and a rainy season: The dry season ranges from October to February while the rainy seasons has two periods–the period from June to September is the main rainy season and some rainy weather period usually from March to May [27]. As of 2019, the health facilities available in the study districts in oromia includes two hospital, 24 health centres and 138 health posts while in Afar one hospital, 5 health centres and 24 health posts. As a common, the most frequently cases observed in the districts are Acute upper respiratory tract infection, Lower respiratory infections such as pneumonia and bronchitis, Diarrheal diseases, Malaria, Urinary tract infections and skin infections. The infant mortality rate for Oromia and Afar region is estimated to be (60 per 1000 live births) and (81 per 1000 live births) respectively, which is higher than the national average of 8 per 1000 live births [28].

### Study population

The study populations were all deceased infants (<1 year of age) and the primary respondent to the VA questionnaire were their mother/primary caregiver who was with the deceased in the period leading to death or a witness to a sudden death or accident.

### Sample size and sampling technique

The sample size estimation used Cochran’s sample size formula for single population proportion using OpenEpi Version 3.3a. The sample size was determined based on the proportion of the most common cause of infant mortality previously identified in the cohort study conducted in Ethiopia [29], which would allowed to yield the largest sample size. Accordingly, the sample size is calculated with an assumption that the proportion of 17.7% for the infant death caused by bacterial sepsis from the overall infant death [29], with a ± 5% precision, 95% level of confidence and a design effect of 1.5. Thus, the calculated sample size was 336 and 10% added for non-respond rate—which resulted a final sample size of 370 infant deaths. This would obtain from 6271 live birth as the national infant death rate was 59 per 1000 live births [9]. Therefore, this survey covered 197,201 populations as calculated by cross-multiplication from the national crude birth rate of 31.8 live births per 1000 inhabitants [28].

A two-stage cluster sampling technique with probability proportional to size (PPS) was employed for this particular study. In the first sampling stage, six districts were selected randomly using lottery method from the two zones (Four districts from West Hararghe zone of oromia region and two from zone 3 of Afar region). The second sampling stage comprised of a random selection of “Kebeles” (a smallest administrative unit) from the randomly selected districts, taking each “kebele” as one cluster. In each Kebele (cluster), all infant deaths occurring were routinely registered by visiting each household every month and questionnaire-based verbal autopsy interview conducted after culturally prescribed mourning period has passed, to a certain extent between 15–30 days of the date of infant death.

#### Data collection instrument and methods

Data was collected by interviewing the mothers or primary caretaker of the deceased infant using a standardize 2014 WHO Verbal Autopsy questionnaire [25]. This questionnaire elicits data on the age and sex of the deceased, diseases, signs and symptoms, as well as circumstances observed preceding death. For this particular study, two questionnaires were used (Death of a child aged under four weeks and Death of a child aged four weeks to 11 years). All the standard questionnaires were translated to local languages “Amharic”and “Afan Oromoo”, and retranslated back into English by relevant experts to verify that the meanings of the questions are retained and checked its consistency.

Data collectors were recruited based on the previous experiences in conducting studies and had good working knowledge in the relevant local language(s). Selection of local data collectors was aimed at ensuring the efficiency of the interview process owing to their inherent knowledge of local customs, expressions and cultural sensitivities. The training in conducting questionnaire-based VA interviews and data collection procedure was provided to the data collectors and supervisors. The questionnaire was pre-tested in a community having similar characteristics to the study area and population. The health extension workers, who stationed at their respective setting, routinely visit each household every month for events; report and notify all infant deaths. After received this, the data collectors conducted VA interviews between 15–30 days of the date of every infant death. The data collection was closely monitored by supervisors and each filled questionnaire was thoroughly checked for completeness as well as consistency and go back to the field for correction when errors were detected.

#### Data quality control

For quality assurance, certain strategies were undertaken to control random and systemic error before, during and after the data collection. Experienced enumerators and supervisors with relevant educational background and language proficiency were recruited. The data collection procedures developed and intensive training was provided for the data collectors and supervisors. Questionnaires were translated to the local language and then back to English in order to maintain its consistency. Pre-testing was also conducted to thoroughly familiarize with data collection instruments and procedures. The targeted respondent who provided data about the deceased was mothers/primary care takers that would provide more credible, reliable and accurate data [25]. The VA interviews conducted after mourning period has passed with shorter recall periods. On-the-spot monitor of data collectors and field editing were carried out in every study area by the assigned field supervisors. The supervisors provided immediate feedback and technical support as needed.

#### Data management and analysis

InterVA-4 model was used for the entire processing and determining cause of death from the collected verbal autopsy data. The collected data was entered into a comma separated variable (.csv) file database in InterVA-4 program, which was prepared a data entry sheet with columns in identical sequence to the questions in the questionnaire. Following the installation procedure outlined in the InterVA-4 user guide [24], the probable causes of death to each deceased infant was determined. As InterVA requires specifying basic epidemiological parameters for malaria and HIV/AIDS prevalence in the population as “Very low”, “low” or “high”, this study leveled as “high” for malaria as the infant death prevalence in Ethiopia for recent years is 1% [30], which being laid on 1:100. For HIV/AIDS, “low” is set as the death prevalence is less than 1% [31], which being pointed at around 1:1000 of all deaths in the population in the context of the guideline.

The InterVA-4 model follows the VA cause of death categories defined in the WHO-2014 standard together with WHO cause codes and corresponding ICD-10 categories, which tend to establish the most likely cause for particular case, with its own likelihood. To estimate cause-specific infant mortality fractions, we summed the likelihood of each causes from every infant death and divided this by the total number of deaths. The data from Excel spreadsheet also exported to SPSS version 23 for statistical analysis. Descriptive statistics were carried out to express data as frequency distribution, means and standard deviations or percentages with 95% confidence intervals. Binary logistic regression was used to analyze the association factors of maternal and infant characteristics among mortality from each of ARI including pneumonia, diarrhea disease, birth asphyxia, prematurity and malaria against all other causes combined. For each outcome, separate models were adjusted to identify factors independently. Factors that had significant level of p<0.2 in bivariate analysis in each model were candidate for the multivariable analysis of conditional logistic regression. In such case, those factors with p<0.05 were considered as statistically significant and had independently association with the outcome variables.

#### Ethical consideration

Before the study begins, an ethical approval was provided by Ethics Review Board of Addis Ababa University—Ethiopian Water Resource Institute and ethical clearance was also received from the research ethics committee of Oromia regional Health Bureau. A formal letter was written by the institution to each study districts administrative as well as health offices and consequently permission was obtained. Interview was carrying out only with full informed consent of the respondent being interviewed. A written informed consent was obtained from each mother/primary caretaker of infants who participated in the study. Before each interview, clear and adequate explanation was given using the participant’s information sheet. Personal data, in particular name, geographical information and contact information about the respondent, is kept and be encrypted to protect privacy and ensure confidentiality. The original paper records were stored in a locked file cabinet, and personal identifiers are removed from study documents and also computer-based files were stored in a password encrypted a laptop to protect the participants’ confidentiality.

## Results

### General characteristics of respondents and study population

During the study period, a total of 362 deceased infants’ mothers/caretakers were interviewed, yielded 97.8% response rate. The highest proportion (90.4%) of the respondents was parents to the deceased infants.’ The mean age (± SD) of the respondent was 27.3 (±4.3) years old. The age distribution of the deceased infants’ mothers/care taker was highest in the age category of 20–34 years old (86.7%). The majority of the respondents were married at 91.2%. More than four-fifths (87.9%) of the respondents were reported not to have been attended formal education at some point in their lives. Almost 88.6% of respondent’s economic activity was home-maker (housewife) in a year prior to infant’s death.

Of the overall recorded infant deaths, about 192 (53.0%) deaths were occurred during neonatal life while 170 (47.0%) in the post-neonatal period. The deceased infant comprised of 205 (56.6%) males and 157 (43.4%) females with an overall sex ratio of 1.31:1. Over half (55.4%) of the infant death were occurred in the wet season. Almost seven out of nine deaths (77.1%) took place outside of health facilities, of which the majority (71.8%) was died at home. A large proportion of the deceased infants reported from districts of the study area in Oromia region at 87.8% (see Table 1).

**Table 1:** Frequency distribution of the respondents and the deceased infant characteristics in Eastern Ethiopia, 2016–18.

Socio-demographic Characteristics	Neonate (<1month) (n = 192)		Post-neonate (1–11 months) (n = 170)		Total (<1 year) (N = 362)	
	n	%	n	%	n	%
**Respondent characteristics**						
Age of the mother/care taker						
<20 Years old	12	3.3	12	3.3	24	6.6
20–34 Years old	169	46.7	145	40.1	314	86.7
≥35years old	11	3.0	13	3.6	24	6.6
Maternal Marital Status						
Married	181	50.0	149	41.2	330	91.2
Single	6	1.7	10	2.7	16	4.4
Divorce	2	0.5	9	2.5	11	3.0
Widowed	3	0.8	2	0.6	5	1.4
Mother’s level of education						
No education	174	48.1	144	39.8	318	87.8
Primary school	15	4.1	23	6.4	38	10.5
Secondary school	3	0.8	2	0.6	5	1.4
College	0	0.0	1	0.3	1	0.3
Maternal Occupational Status						
Home maker (Housewife)	176	48.6	145	40.1	321	88.7
Employed	4	1.1	12	3.3	16	4.4
Mainly unemployed	12	3.3	13	3.6	25	6.9
**Deceased Infant Characteristics**						
Infant sex						
Male	117	32.3	88	24.3	205	56.6
Female	75	20.7	82	22.7	157	43.4
Seasons of death						
Wet season	107	29.6	94	26.0	201	55.5
Dry season	85	23.5	76	21.0	161	44.5
Place of death						
At home	131	36.2	129	35.6	260	71.8
At health facilities	55	15.2	28	7.7	83	22.9
On the route to health facility	6	1.7	8	2.2	14	3.9
Other places	0	0.0	5	1.4	5	1.4
Death by districts^⊥^						
Districts in Afar region	20	5.5	24	6.6	44	12.2
Districts in Oromia region	172	47.5	146	40.3	318	87.8

^⊥^ Districts in Afar region (Amibara and Awash Fentale), Districts in Oromia (Meiso, Chiro, Tulo and Gemechis)

### Specific cause of infant deaths: Result from verbal autopsy (interVA-4)

Based on the result of the InterVA-4 model, the mean (± SD) of the likelihood value for the assigned cause of death across cases was 96.5% (±4.8%). The likelihood rate across cases varied with the range between 85–100%. In this analysis, the probable causes of infant deaths were classified into an age group of neonatal (<1month) and post-neonatal (1-11months). The analysis revealed that there were considerable major differences in the causes of infant death structure by age group.

#### Neonatal cause of death (n = 192)

Across all the study area, nearly half of neonatal deaths (47.0%) occurred within 24 hours of birth, and about 10.4% die more than 24 hours after birth but within 48 hours from birth. For a further 14.6% deaths occurred more than 48 hours from birth but within the first week of life and for the rest 28.0% deaths were after the first week, but within first 28 days.

Among neonatal deaths, the three major causes of deaths were neonatal pneumonia (33.3%), followed by birth asphyxia (30.9%) and prematurity (13.9%). The fourth leading cause of death was meningitis and encephalitis which was responsible for 5.2 percent of death under this age group. Congenital malformation and neonatal sepsis were found to be another cause of neonatal mortality with 4.5 percent and 3.7 percent respectively. Diarrhea diseases caused about 2.0 percent of neonate death and for the remaining 1.8 percent no cause could be determined which concluded under other and unspecified neonatal cause of death.

#### Post-neonatal cause of death (n = 170)

In the post-neonatal age group, the leading cause of death was acute respiratory infection including pneumonia, which is responsible for 44.2% of all deaths in this age category. Close to one-third (32.5%) of death were due to diarrheal diseases which accredited to the second largest cause of deaths and malaria positioned at the third major cause of death, which accounted for one of every ten deaths (9.2%).

The result revealed that about 2.8 percent of deaths were due to severe malnutrition. The other illness that attributed to death under this age group was measles, which took a toll of 2.2 percent followed by pulmonary tuberculosis (1.6%) and non-obstetric sepsis (1.1%). Few deaths were ascertained due to HIV/AIDs related and meningitis and encephalitis each accounted for 0.6% of death. Deaths due to accidents found to be 2.4%, typically from contact with venomous animals and plants (1.8%) and accidental poisoning and exposure to noxious substance (0.6%). The remaining (0.6%) appeared to be other and unspecified infectious disease cause of death.

#### Overall cause of infant death (n = 362)

Of the total infant did, the findings indicate the highest mortality load among infants was diseases of respiratory system, particularly acute respiratory infection including pneumonia in post-neonate and neonatal pneumonia, which accounted for 20.7% and 17.7% respectively and causing in a combination of 38.4 percent infant deaths in the study area.

The mortality risk due to birth asphyxia was the next leading cause of deaths, accounted for 16.4% of infant death. Almost one-sixth (16.3%) of the infant were died as a result of diarrheal diseases. About 7.4% of deaths were associated with prematurity. Deaths due to malaria, meningitis and encephalitis, and sepsis appeared to be another identified cause of death conditions that contributed to 4.3%, 3.0%, and 2.5% of infant death, respectively. Moreover, congenital malformation (2.4%), severe malnutrition (1.3%) and measles (1.0%) were also observed as causes that directly lead to infant deaths. Pulmonary tuberculosis (0.8%) and HIV/AIDS related death (0.3%) were the least ranked conditions among the deceased infant, and further 1.1% was causes due to accidents.

As per consolidated into a broad WHO cause category, Infectious and parasitic diseases causes exhibited 46.7 percent—with a high burden on post neonate age group, followed by neonatal cause of death (45.9%), nutritional and endocrine disorder (1.3%), external causes of death (1.1%) and the remaining (1.3%) were reportedly unknown cause (see Table 2).

**Table 2:** Cause-specific infant mortality fraction by WHO VA category and age group in Eastern Ethiopia, 2016–18.

Code	Causes of Infant Death *(WHO VA cause category)*	Frequency %(CI,95%) Distribution of Infant Death		Total % (CI, 95%)
		Neonate	Post Neonate	
**01**	**Infectious and parasitic diseases**	**3.8(1.9–5.9)**	**43.1(38.1–48.2)**	**46.9(41.8–52.1)**
	01.01 Sepsis	-	0.5(0.0–1.3)	0.5(0.0–1.3)
	01.02 Acute respiratory infection, including pneumonia	-	20.7(16.5–24.9)	20.7(16.5–24.9)
	01.03 HIV/AIDS related death	-	0.3(0.0–0.8)	0.3(0.0–0.8)
	01.04 Diarrheal diseases	1.0(0.03–2.2)	15.3(11.5–18.9)	16.3(12.5–20.1)
	01.05 Malaria	-	4.3(2.3–6.5)	4.3(2.3–6.5)
	01.06 Measles	-	1.0(0.03–2.2)	1.0(0.03–2.2)
	01.07 Meningitis and encephalitis	2.8(1.1–4.5)	0.2(0.0–0.7)	3.0(1.3–4.8)
	01.08 Pulmonary tuberculosis	-	0.8(0.0–1.7)	0.8(0.0–1.7)
**03**	**Nutritional and endocrine disorders**	**-**	**1.3(0.13–2.5)**	**1.3(0.13–2.5)**
	**03.01 Severe malnutrition**	-	1.3(0.13–2.5)	1.3(0.13–2.5)
**10**	**Neonatal causes of death**	**45.9(40.7–51.0)**	**-**	**45.9(40.7–51.0)**
	10.01 Prematurity	7.4(5.1–10.7)	-	7.4(5.1–10.7)
	10.02 Birth asphyxia	16.4(12.6–20.2)	-	16.4(12.6–20.2)
	10.03 Neonatal pneumonia	17.7(13.8–21.6)	-	17.7(13.8–21.6)
	10.04 Neonatal sepsis	2.0(0.6–3.4)	-	2.0(0.6–3.4)
	10.05 Congenital malformation	2.4(0.8–3.9)	-	2.4(0.8–3.9
**12**	**External causes of death**	**-**	**1.1(0.03–2.2)**	**1.1(0.03–2.2)**
	12.01 Contact with venomous animals and plants	-	0.8(0.0–1.7)	0.8(0.0–1.7)
	12.02 Accidental poisoning and exposure to noxious substance	-	0.3(0.0–0.9)	0.3(0.0–0.9)
**99**	**Cause of death unknown**	**1.0(0.03–2.2)**	**0.3(0.0–0.9)**	**1.3(0.13–2.5)**
	**Total deaths**	**50.7(45.6–55.9)**	**45.8(40.7–50.9)**	**96.5**

### Pattern of associated factors with specific-causes of deaths against all-causes of death

Independent factors in multivariable analysis have been identified through pattern of selected factors associated with each leading specific-causes of infant death (ARTI including pneumonia, diarrheal death, birth asphyxia, prematurity and malaria) as compared to those infant who died from all other cause of death in combined.

Result from ARI-specific mortality model showed that, infant mortality from Acute respiratory infection, including pneumonia shows a strong significant association with younger maternal age (<20 years old), deaths out of heath facilities and unmarried women than all other causes of infant death. Lower maternal age (<20 Years old) was almost 5 times more likely to die of Acute respiratory infection, including pneumonia than all other causes of infant death combined (*P* = 0.001, AOR: 4.82, 95% CI: 1.88, 12.3). Infants being died out of health facilities were also associated with nearly 3 times higher risk of ARTI death as compared to mortality from all other causes (*P* = 0.007, AOR: 2.85, 95% CI: 1.33, 6.12). Significant interaction were observed for those died infants with unmarried mothers, but resulted in lower chance (*P* = 0.041, AOR: 0.46, 95% CI: 0.22, 0.97) of ARTI-related death than those who died from other all-causes (see Table 3).

**Table 3:** Multivariable logistic regression analysis of factors associated with cause-specific mortality against all other cause of infant death in Eastern Ethiopia, 2016–18.

Factors	Adjusted odds Ratio (95% CI)				
	Model 1	Model 2	Model 3	Model 4	Model 5
**Maternal Age**					
<20 Years old	4.82 (1.88, 12.3)*	0.14 (0.02, 1.11)			1.43 (0.17, 11.8)
20–34 Years old	1.03 (0.43, 2.50)	1.14 (0.36, 3.64)			4.44 (1.22, 16.2)*
≥35years old	1	1			1
**Marital status**					
Unmarried	0.46 (0.22,0.97)*	0.85 (0.33, 2.18)	6.92(0.91, 52.4)		
Married	1	1	1		
**Mother’s education**					
No education			0.59(0.19, 1.84)	0.24(0.03, 1.85)	
Educated			1		
**Occupational status**					
Housewife			0.39(0.11, 1.37)		
Others			1		
**Infant Age**					
Post-neonate	1.40(0.90, 2.18)	15.5(6.35, 37.8)*			
Neonate	1	1			
**Sex of Infant**					
Male		1.21(0.65, 2.27)	0.50(0.27, 0.92)*		
Female		1	1		
**Seasons of death**					
Wet seasons		2.38(1.28, 4.44)*			0.22 (0.06, 0.82)*
Dry seasons		1			1
**Place of death**					
Outside of health facilities	2.85 (1.33,6.12)*	1.49 (0.46, 4.77)	0.34(0.17, 0.67)	0.24(0.10, 0.57)*	
Health facilities	1	1	1	1	
**Administration Division**					
Districts in Afar region					4.08 (1.35, 12.4)*
Districts in Oromia region					1

Gray Shade: Factors that hadn’t p-value of <0.2 from bivariate analysis & not eligible in multivariable

Model 1: Association of factors with deaths due to **ARTI** versus all other causes of death

Model 2: Association of factors with deaths due to **diarrhea** versus other cause of death

Model 3: Association of factors with deaths due to **birth asphyxia** versus other cause

Model 4: Association of factors with deaths due to **prematurity** versus other cause of death

Model 5: Association of factors with deaths due to **malaria** versus other cause of death

*statistically significant at p<0.05

Analysis from diarrhea-specific mortality model indicated that post-neonates period and wet seasons have more pronounced significant association with diarrhea death than all-cause of death. The post-neonatal period had almost 16 times higher risk of diarrhea death than non-diarrheal deaths (*P* = 0.000, AOR: 15.5, 95% CI: 6.35, 37.8). Wet seasons had significantly 2 times higher chance of infant death due to diarrhea than other cause of death (*P* = 0.006, AOR: 2.38, 95% CI: 1.28, 4.44) (see Table 3).

Infant mortality from birth asphyxia shows a statistical significant relationship, but less likely to occur, with male sex (*P* = 0.039, AOR: 0.52, 95% CI: 0.28, 0.97) and being died out of heath facilities (*P* = 0.002, AOR: 0.32, 95% CI: 0.16, 0.66), as compared to mortality from all other causes. Infant died out of health facilities was the only associated factor with prematurity death, but lower odds (*P* = 0.001, AOR: 0.24, 95% CI: 0.10, 0.57) (see Table 3).

Infant who died from malaria is more closely associated with age of mothers between 20–35 years old and infant who resided in districts of Afar region. Infant with age of mothers between 20–35 years old were 4 times (*P* = 0.024, AOR: 4.44, 95% CI: 1.22, 16.2) higher to die of malaria than malaria-unrelated death. Similarly, infant who resided in districts of Afar region were 4 times higher to die of Malaria than non-malaria death (*P* = 0.013, AOR: 4.08, 95% CI: 1.35, 12.4). Significant association is observed on the risk of death from malaria in wet season, but the likelihood of dying resulted less (*P* = 0.024, AOR: 0.22, 95% CI: 0.06, 0.82) (see Table 3).

## Discussion

The exact cause of death is ascertained by postmortem autopsy [32]. However, this is not practically applicable in most developing countries and, as an alternative Verbal Autopsy has become one of the major sources of data for causes of death, which also used for identification of major health problems and comparison of local and national mortality ratio differences [33]. Most of the published on cause of death using verbal Autopsy data was interpreted by physician review, and this approach is time-consuming, costly and inconvenient [22, 34]. In recent years, the electronically determination of death cause has been introduced and provide an analysis solution that is more convenient, consistent, and rapid ways to interpret VA data [34]. In our study context, InterVA-4 model is preferred to use for assigning cause of infant death as this model is relatively better than other available models (SmartVA/Tarrif, InSilicoVA), particularly for neonate and children death, when contrasting in terms of the degree of chance corrected concordance (CCC), CCVA (Computerized coding of verbal Autopsy) population Accuracy, PCVA (Physician-certified verbal autopsy) performance [35, 36]. In view of that, the present study was undertaken to determine the cause of death using InterVA-4 model and analyze the pattern of associated factors between the most leading cause-specific mortality against all-causes of death.

In this study, many different causes of infant death were observed, from infection to birth defects or accidents. The study result revealed that the proportion of death in the neonatal stage was higher in contrast to post-neonatal period. Similar view was seen in other studies [29, 37–39]. These findings clearly proven as neonatal period is the most vulnerable time for a child death [40]. The most common causes of infant death in the neonatal period are different from those that occur in the post-neonatal. Analyses in the interpretation of specific causes of infant mortality indicated that neonatal pneumonia, birth asphyxia and prematurity were the three major causes of deaths during the neonatal period. Regardless of proportional differences, Birth Asphyxia and Prematurity as a common appeared to be leading causes, which have similarity to those of studies conducted in some parts of Ethiopia [10, 41–43]—and other developing countries [37]. Some studies described Infections diseases (sepsis, pneumonia, meningitis, tetanus and diarrhea) are the leading cause [10, 41, 44], which had methodologically and/or application tools differences with this study. Evidences from several studies conducted in Ethiopia reported that Prematurity, Birth asphyxia, and Neonatal sepsis were the leading causes for neonatal death [29, 45]. These results are consistent to the present study except neonatal sepsis as the proportion of deaths due to sepsis appeared to be less in the study area.

Neonatal Pneumonia is a devastating condition [46] and remained one of the most cause of death in Ethiopians children [47]. It is a serious respiratory infectious disease in a neonate which can be prevented by simple measures such as treatment of maternal infections, careful obstetric care and general infection control measures in neonatal facilities. The higher risks of death from birth asphyxia might be due to lower institutional delivery coverage and mother’s education is essential which might be expected in large reduction in some circumstances. The burden of prematurity seemed to be mostly due to caregivers failed to recognize the danger signs related to prematurity and its consequences. It has been recognized that behaviors that encourage a healthy pregnancy plays an important role in preventing premature labour.

In the post-neonatal period, acute respiratory infection including pneumonia, diarrheal diseases and malaria were the most common causes of death. Despite means of verification and proportional differences, these results showed a similar pattern with the finding of other studies within nationwide [47]. Acute respiratory infection including pneumonia and Diarrheal diseases were mostly appeared as a common cause during post-neonate period, which showed in other inland studies [29, 47], and somewhere else [37–39, 48]. The present study indicated as Malaria was the third major cause of death in this age category, which are quite different from deaths identified in other studies of the country as bacterial sepsis were observed in one of the top three causes of post-neonate death [29] as well as malnutrition [49]. Similar view was observed in studies conducted in other countries [37–39]. As per consolidation into a broad cause category, Infectious and parasitic disease causes were the leading causes of death during the post-neonatal period, which is in agreement with previous studies [29, 47].

In overall infant death, acute respiratory infection including pneumonia, birth asphyxia and diarrhea disease were the most common caused in the present study. Apart from magnitude and rank order disparity, the findings of acute respiratory infection including pneumonia and diarrhea disease as a major probable cause of infant death has similarity with those of the results reported in other parts of the country [10, 47, 49–51], and elsewhere [37, 52]. It can be observed that Prematurity, Malaria, Meningitis and encephalitis, Sepsis and Congenital malformation were other condition resulting deaths of Eastern Ethiopia children, which are more or less the same with causes occurred in most parts of developing countries [48, 53, 54].

The higher risk of death due to acute respiratory infection in the current study might be due to less awareness of the disease transmission and prevention, weak case management in health services, less understanding on early recognition of pneumonia cases and inappropriate care-taking by the parents [55]. Acute respiratory infection including pneumonia may exist due to lack of adequate through and cross ventilation system in the dwelling [56], overcrowding or suffocation in bedroom and weak maternal health, which have a potential favors to transmit the infection [57].

Diarrheal diseases were another most important cause of infant death as reflected by the facts that almost one-sixth of infants reported to have been dead. Infant and children are more likely to die due to lack of safe water and sanitation, along with poor hygiene practices, that result in deteriorative synergy that leads to diarrheal disease [58]. Health seeking behavior such as poor early sough of health care, less measure to diarrhea disease management including oral rehydration therapy (ORT) for rehydration were responsible for diarrhea-related mortality [59]. Other deaths due to severe malnutrition, Measles, Pulmonary tuberculosis and HIV/AIDS appeared to be tribulation of infant’s survival on the basis of the present study result. However, deaths due to tetanus were not observed. This disease was remained as a noticeable death causes of Ethiopian children [8, 47, 51, 60]. The nonappearance of death due to tetanus is most likely associated with being controlled through tetanus toxoid (TT) routine immunization program. Likewise, the less infant death from malnutrition attributed to high-impact nutrition interventions delivery through an integrated package by government and non-governmental organization.

The present study also compares the patterns of factors associated with the leading specific cause of infant death against all other cause of death. Our finding revealed that the patterns of association among each cause-specific mortality were quite different when evaluate with that of its comparison group (all-cause of death in combined). Infants with lower maternal age (<20 years old) were five-fold an increased risk of death from ARI, including pneumonia in contrast to infant with higher maternal age (≥35 years old). This finding suggested as lower maternal age group has largely contributed to multiplying ARI-specific infant mortality risk than all other causes of death. Studies revealed that children having adolescent mother was a strong risk factor for ARI-related death [55, 61]. This could be possibly due to the assumption that lower aged mothers tend to have lower experience in pneumonia-related health care of children and, therefore, greater susceptibility to severe forms of infections that could be drives to mortality. The risk of infant death due to ARI again rises with those infant who died out of heath facilities, which shows three-fold at an increase risk of ARTI, including pneumonia death than ARTI-unrelated infant death. This, in turn, indicated the contribution of death that occurred at home, which might probably due to lack or delay in seeking appropriate medical care utilization. This showed to be highly increases risk of dying from pneumonia than died from other causes [62].

Another important finding was that, the post-neonatal period is the most predominant factor in contributing to the greater elevated by nearly sixteen times of diarrheal death risk than the corresponding risk of all-causes of death. The strong influence of this age period on diarrhea-related death had been examined in other studies [63, 64]. Our study also revealed that wet seasons are strong significant risk factors by twice for diarrhea-related death than all other causes. The study conducted in India and Mexico where largely confirmed that deaths caused by diarrhea are strongly linked to seasons of death with more pronounced during cooler months [64, 65]. This might be due to the fact that diarrhea diseases had more potential to transmit in wet seasons, and increase its incidence that initiated the series of events leading to death.

Our study has also found that being male sex is significantly association with death from birth asphyxia; however, the risk of dying has been less likely as compared to other cause of death. This linkage could be attributed to behavioral, biological, socio-cultural, and genetic factors, which needs further study. We also observed that male is the most predominance in dying from birth asphyxia; similar finding was observed in various studies [66, 67]. Our analysis indeed showed significant association in place of death that occurred out of health facilities for birth asphyxia against other causes; though the likelihood of its effect is less. The study similarly demonstrated that place of death in out of health facilities are the only significant factor with lower chance for premature death as compared to all-cause mortality.

It has been also observed that infant mortality resulted from malaria significantly related to infant’s mothers/care takers age group of 20–34 years with four times highest-risk chance in contrast to maternal age ≥35 years old, as evaluated from that of all-causes of death. A study on the link between early childhood mortality and malaria in Malawi reported that children were significantly at greater risk if the mother was lower age relative to older age [68]. The geographical administration division are appears to be statistically significant to the risk of malaria-specific infant death. Infants resided in the study area of districts in Afar region were four times substantial raised risk of malaria deaths as compared to districts in Oromia region, as evaluated against all other causes. A study in Burkina Faso has demonstrated the significant variations in all-cause and malaria-specific mortality across village clusters [69]. The differences by geographical administrative division might be due to variability in socio-demographic and economic factors, the prevalence of malaria incidence, weather condition, and variability of quality of health care service. However, further studies are needed to better understand for these disparities. Significant interaction was seen between wet seasons of infant death with the risk of malarial-specific death. However, this risk is less likely as observed in comparison to the all-cause of death. A study in West Africa indicated that malaria-specific infant death had significant seasonal trends and higher in the rainy seasons [70].

### Strength and limitation

The strengths of this study include that it used validated verbal autopsy tool and model to determine all cause of infant death with quantified degrees of certainty. Longitudinal survey covering with large geographic area, routinely enumeration of reported and notified deceased infants as well as data quality procedure is also strong side of the study. Despite a very few studies available in the analysis of cause of infant death at population level, this study provided useful information for building evidence based health policy, better insights in planning the best solutions, and input for policy-makers in taking appropriate measures.

Despite the verbal autopsy used as an alternative method in determining the cause of death, there was recognized limitation on the tools and methods which might have influenced in the determination of cause of death. The InterVA model assign cause of death only based on the present of sign and symptoms, disregarding them entirely when there are absent. InterVA marginalize over that symptom if the respondent does not report a symptom. The respondent who replied the VA questionnaire about the deceased infant might not be provided full data for the reason of sorrow and grief. Drawback due to inability of the respondents to recognize, recall and trace signs and symptoms of diseases that leads to death prior to the deceased. In order to minimize this, linguistic and ethnographic work as well as skilled interviewers were recruited and adequately trained to capture data.

### Conclusion

The study concludes that the majority of infant deaths existed in the present study were as a result of diseases and conditions that are readily preventable or treatable cause. Majority of infant deaths were occurring in the first month of life and the leading causes of infant mortality appeared to be acute respiratory infection including pneumonia, birth asphyxia and Diarrheal diseases. The age categories have a broad effect across many causes of death, as having been revealed that Neonatal pneumonia, birth asphyxia and prematurity were the major causes of deaths during the neonatal period, while acute respiratory infection including pneumonia, Diarrheal diseases and Malaria appeared to be in post-neonate period. The patterns of significant associated factors across cause-specific mortality against all-cause of death were dissimilar. Thus, more efforts require on maternal and child health program with proven preventive interventions emphasizing on the most common cause of infant death. Attention should also be given to those factors that make the specific death more likely to occur. Proper screening for infections in pregnant women, encouraging institutional delivery, Immunization, provision of safe water and sanitation with adoption of hygiene practices, promotes cross and through ventilation system into dwelling and ensuring appropriate preventive and curative care for infants should be strengthen.

## Supporting information

S1 File Household level verbal autopsy questionnaire. (DOCX)

S2 File Database for the cause of infant mortality and associated factors. (SAV)

## Abbreviations

ARTI: Acute Respiratory Tract Infection
CI: Confidence Interval
CSV: Comma Separated Variable
EIWR: Ethiopian Institute of Water Resources
ICD: International Classification of Disease
SD: Standard Deviation
SDG: Sustainable Development Goal
VA: Verbal Autopsy
WHO: World Health Organization
