# Supplementary material for: Causes of infant deaths and patterns of associated factors in Eastern Ethiopia: Results of verbal autopsy (InterVA-4) study
Source: PLoS One. 2022 Aug 4;17(8):e0270245. doi: 10.1371/journal.pone.0270245 (PMC9352103; doi:10.1371/journal.pone.0270245)
Supplement: S1 File — (DOCX) [file pone.0270245.s001.docx]

**ADDIS ABABA UNIVERSITY**

**ETHIOPIAN INSTITUTE OF WATER RESOURCES**

**HOUSEHOLD LEVEL VERBAL AUTOPSY QUESTIONNAIRE**

**INFORMATION SHEET AND CONSENT FORM**

Good Morning/Afternoon!

My name is………………………….………… I’m a data collector for the study associated with cause of infant mortality. This study is organized by Addis Ababa University, Institute of Water Resources. I would like to ask you about the recent illness of your child that led to his/her death.

All the information that you provide will be kept confidential, and will only be used to understand the causes of infant deaths with their contributing factors in your area. This information will be used to improve the health program for our community to decrease future deaths. Your participation is voluntary and you are not obliged to answer any question you do not wish to answer. If you are not comfortable with the interview, you have full right to refuse or participate in the study at any time you want. But your honest response will contribute to generate information, which can be used to improve the health problems associated with mortality. Your name will be written on our forms so we can recognize what you said and contact you in the future if necessary. However, when the data are put together with those from other people, nobody else will have access to your name, if you prefer so.

We would greatly appreciate your help in responding to this interview. The interview will take about ***25-30 minutes***. Would you be willing to participate?”

1. If yes, continue to interview

2. If no, skip to the other households

**Instruction to the Interviewers**:

- For the deceased neonate (<1month of age), use Verbal Autopsy Questionnaire (I)
- For the deceased post-neonate ( 1-11months of age), use Verbal Autopsy Questionnaire (II)

**
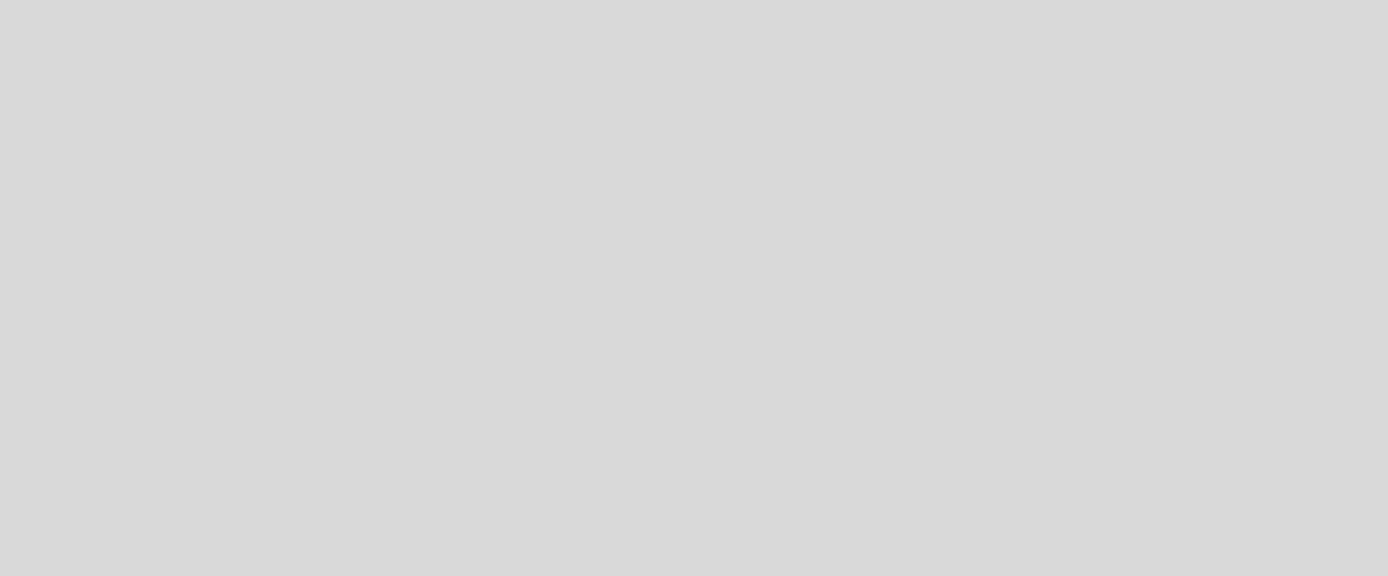
VERBAL AUTOPSY**

**QUESTIONNAIRE (I)**

**Death of a child aged <1month**

| **NO.** | **QUESTIONS AND FILTERS** |  | **ANSWER** |  | |  | **SKIP** | |  |
| --- | --- | --- | --- | --- | --- | --- | --- | --- | --- |
| 0A100a | Is this a region of high HIV/AIDS prevalence? | YES | |  | |  |  |  |  |
|  |  | NO | |  | |  |  |  |  |
| 0A100b | Is this a region of high malaria prevalence? | YES | |  | |  |  |  |  |
|  |  | NO | |  | |  |  |  |  |
| **SECTION 1. INFORMATION ON THE DECEASED** | |  |  |  | |  |  |  |  |
| 1A100a | What was the first or given name(s) of the deceased?  _________________________________________________________ | | |  | |  |  |  |  |
|  |  |  |  |  | |  |  |  |  |
| 1A100b | What was the surname (or family name) of the deceased?  __________________________________________________________ | | | | |  |  |  |  |
|  |  |  |  |  |  |  |  |  |  |
| 1A110 | What was the sex of the deceased? |  | MALE | |  |  |  | |  |
|  |  |  |  |  |  |  |  |  |  |
|  |  | FEMALE | | |  |  |  |  |  |
| 1A200 | Is the date of birth known? | YES | | |  |  | ➡ |  |  |
|  |  | NO | | |  |  |  | 1A220 |  |
|  |  | Refuse to answer | | |  |  | ➡ | 1A220 |  |
| 1A210 | When was the deceased born? |  | DAY | |  |  |  |  |  |
|  |  |  |  |  |  |  |  |  |  |
|  |  |  | MONTH | |  |  |  |  |  |
|  |  |  | YEAR | |  |  |  |  |  |
|  |  |  |  | |  |  |  |  |  |
| 1A220 | Is the date of death known? | YES | | |  |  | ➡ |  |  |
|  |  | NO | | |  |  |  | AAAA |  |
|  |  | Refuse to answer | | |  |  | ➡ | AAAA |  |
| 1A230 | When did (s)he die? |  | DAY | |  |  |  |  |  |
|  |  |  | MONTH | |  |  |  |  |  |
|  |  |  | YEAR | |  |  |  |  |  |
|  |  |  |  | |  |  |  |  |  |
| AAAA | Enter neonate's age in days |  | DAYS | |  |  |  |  |  |
|  |  |  |  |  |  |  |  |  |  |
|  |  |  |  | |  |  |  |  |  |
| AAAA | Enter neonate's age in hours |  | HOURS | |  |  |  |  |  |
|  |  |  |  | |  |  |  |  |  |
| AAAA | Enter neonate's age in minutes |  | MINUTES | |  |  |  |  |  |
|  |  |  |  | |  |  |  |  |  |
| 1A500 | What was her/his citizenship / nationality? | Citizen at birth | | |  |  |  |  |  |
|  |  | Naturalized citizen | | |  |  |  |  |  |
|  |  |  | Foreign national | |  |  |  |  |  |
|  |  | Don’t know | | |  |  |  |  |  |
| 1A510 | What was her/his ethnicity? _________________________________________ | | | | |  |  |  |  |
|  |  |  |  |  |  |  |  |  |  |
| 1A520 | What was her/his place of birth? _______________________________________ | | |  | |  |  |  |  |
|  |  |  |  |  | |  |  |  |  |
| 1A530 | What was her/his place of residence? ( – if applicable) _______________ | | | | |  |  |  |  |
|  |  |  |  |  |  |  |  |  |  |
| 1A550 | Where did death occur?(specify country, province, district, village)  ____________________________________ | | | | |  |  |  |  |
|  |  |  |  |  |  |  |  |  |  |
| 1A560 | Where did the deceased die? | | Hospital | |  |  |  |  |  |
|  |  |  | Other health facility | |  |  |  |  |  |
|  |  |  | Home | |  |  |  |  |  |
|  |  |  | On route to facility or  hospital | |  |  |  |  |  |
|  |  |  | Other | |  |  |  |  |  |
|  |  |  | Don’t know | |  |  |  |  |  |
|  |  |  | Refuse to answer | |  |  |  |  |  |
| 1A620 | What was the name of the father? |  |  | |  |  |  |  |  |
|  | Surname: ________________________ Name: ______________________ | | | | |  |  |  |  |
| 1A630 | What was the name of the mother? | | | | |  |  |  |  |
|  | Surname: ________________________ Name: ______________________ | | | | |  |  |  |  |

| **SECTION 2. VITAL REGISTRATION AND CERTIFICATION** | | | | | | | | | | | | | |  |
| --- | --- | --- | --- | --- | --- | --- | --- | --- | --- | --- | --- | --- | --- | --- |
| 1A700 | | Death registration number/certificate __________________________________________________________ | | | | | | | | | |  | |  |
|  | |  |  |  |  |  |  |  |  |  |  |  | |  |
| 1A710 | | Date of registration | DAY | | | | |  | | | |  | |  |
|  | |  |  |  |  |  |  |  |  |  |  |  | |  |
|  | |  | MONTH | | | | |  | | | |  | |  |
|  | |  | YEAR | | | | |  | | | |  | |  |
|  | |  |  | | | | |  | | | |  | |  |
| 1A720 | | Place of registration  _____________________________________________________________ | | | | | | | | | |  | |  |
|  | |  |  |  |  |  |  |  |  |  |  |  | |  |
| 1A730 | | National identification number of deceased _____________________________________________________________ | | | | | | | | | |  | |  |
|  | |  |  |  |  |  |  |  |  |  |  |  | |  |
| **SECTION 3. INFORMATION ON THE RESPONDENT AND BACKGROUND ABOUT INTERVIEW** | | | | | | | | | | | | | |  |
|  | |  |  | | | | |  | | | |  | |  |
| 2A100 | | What is the name of VA respondent? |  | | | | |  | | | |  | |  |
|  | | Surname: _______________________ Name: __________________________ | | | | | | | | | |  | |  |
| 2A110 | | What is the respondent's relationship to the deceased? | Parent | | | | |  | | | |  | |  |
|  | |  | Child | | | | |  | | | |  | |  |
|  | |  | Other family member | | | | |  | | | |  | |  |
|  | |  | Friend | | | | |  | | | |  | |  |
|  | |  | Health worker | | | | |  | | | |  | |  |
|  | |  | Public official | | | | |  | | | |  | |  |
|  | |  | Another relationship | | | | |  | | | |  | |  |
| 2A115 | | Did the respondent live with the deceased in the period  leading to her/his death? | YES | | | | |  | | | |  | |  |
|  | |  | NO | | | | |  | | | |  | |  |
|  | |  | Don’t know | | | | |  | | | |  | |  |
|  | |  | Refuse to answer | | | | |  | | | |  | |  |
| 2A120 | | Name of VA interviewer    Surname: ____________________________ Name:_______________________ | | | | | | | | | |  | |  |
| 2A130 | | Time at start of interview | hh:mm 24h | | | | ___:___ | | | | |  | |  |
|  | |  |  |  |  |  |  |  |  |  |  |  | |  |
| 2A130 | | Time at end of interview | hh:mm 24h | | | | ___:___ | | | | |  | |  |
|  | |  |  | | | |  | | | | |  | |  |
| 2A140 | | Date of interview | DAY | | | |  | | | | |  | |  |
|  | |  | MONTH | | | |  | | | | |  | |  |
|  | |  | YEAR | | | |  | | | | |  | |  |
| 2A150 | | Did the respondent give consent? | YES | | | |  | | | | |  | |  |
|  | |  | NO | | | |  | | | | |  | |  |
| 3A280 | | During which season did (s)he die? | Wet | | | |  | | | | |  | |  |
|  | |  | Dry | | | |  | | | | |  | |  |
| 3A310 | | Did (s)he die suddenly? | YES | | | |  | | | | |  | |  |
|  | |  | NO | | | |  | | | | |  | |  |
|  | |  | Don’t know | | | |  | | | | |  | |  |
|  | |  | Refuse to answer | | | |  | | | | |  | |  |
| 3A3100 | | What age of the respondent (in full years) |  | | | |  | | | | |  | |  |
| 3A3101 | What was her/his marital status? | | Single | | | |  | | |  |  | |  |  |
|  |  |  | Married | | | |  | | |  |  | |  |  |
|  |  |  | Life partner | | | |  | | |  |  | |  |  |
|  |  |  | Divorced | | | |  | | |  |  | |  |  |
|  |  |  | Widowed | | | |  | | |  |  | |  |  |
|  |  |  | Too young to be  married | | | |  | | |  |  | |  |  |
|  |  | | Don’t know | | | |  | | |  |  | |  |  |
|  |  | | Refuse to answer | | | |  | | |  |  | |  |  |
| 3A 3101 | What was the date of marriage? | |  | | | DAY |  | | |  |  | |  |  |
|  |  |  |  | | |  |  |  |  |  |  | |  |  |
|  |  |  |  | | | MONTH |  | | |  |  | |  |  |
|  |  |  |  | | | YEAR |  | | |  |  | |  |  |
| 3A3102 | What was her/his highest level of schooling? | | | No formal education | | |  | | |  |  | |  |  |
|  |  |  |  | Primary school(1-8 Grade) | | |  | | |  |  | |  |  |
|  |  |  |  | Secondary school (9-12 Grade) | | |  | | |  |  | |  |  |
|  |  |  |  | Higher then secondary school | | |  | | |  |  | |  |  |
|  |  | | | Don’t know | | |  | | |  |  | |  |  |
|  |  | | | Refuse to answer | | |  | | |  |  | |  |  |
| 3A3103 | Was (s)he able to read and write? (select 'yes' also if only one of either reading or writing is know to the respondent) | | | | YES | |  | |  | | | | |  |
|  |  |  |  |  | NO | |  | |  | | | | |  |
|  |  |  |  |  | Don’t know | |  | |  | | | | |  |
|  |  |  |  |  | Refuse to answer | |  | |  | | | | |  |
| 3A3104 | What was her/his economic activity status in year prior to death? | | | | Mainly  unemployed | |  | |  | |  | |  |  |
|  |  |  |  |  | Mainly employed | |  | |  | |  | |  |  |
|  |  |  |  |  | Home-maker | |  | |  | |  | |  |  |
|  |  |  |  |  | Pensioner | |  | |  | |  | |  |  |
|  |  |  |  |  | Student | |  | |  | |  | |  |  |
|  |  |  |  |  | Other | |  | |  | |  | |  |  |
|  |  |  |  |  | Don’t know | |  | |  | |  | |  |  |
|  |  |  |  |  | Refuse to answer | |  | |  | |  | |  |  |
| 3A3105 | What was her/his occupation, that is, what kind of work does (s)he mainly do?  ________________________________________________ | | | | | | | |  | | | | |  |
|  |  |  |  |  |  |  |  |  |  |  |  |  |  |  |
|  |  |  |  |  |  |  |  |  |  |  |  |  |  |  |



| **SECTION 4. GENERAL SIGNS AND SYMPTOMS ASSOCIATED WITH FINAL ILLNESS** | | | | | | | |  |  |  |
| --- | --- | --- | --- | --- | --- | --- | --- | --- | --- | --- |
| 3B100 | Did (s)he have a fever? | YES |  | | ➡ |  | |  |  |  |
|  |  | NO |  | |  | 3B130 | |  |  |  |
|  |  | Don’t know |  | | ➡ | 3B130 | |  |  |  |
|  |  | Refuse to answer |  | | ➡ | 3B130 | |  |  |  |
| 3B110 | How many days did the fever last? | DAYS |  | |  |  | |  |  |  |
| 3B130 | Did (s)he have a cough? | YES |  | |  |  | |  |  |  |
|  |  | NO |  | |  |  | |  |  |  |
|  |  | Don’t know |  | |  |  | |  |  |  |
|  |  | Refuse to answer |  | |  |  | |  |  |  |
| 3B180 | Did (s)he have any breathing problem? | YES |  | |  |  | |  |  |  |
|  |  | NO |  | |  |  | |  |  |  |
|  |  | Don’t know |  | |  |  | |  |  |  |
|  |  | Refuse to answer |  | |  |  | |  |  |  |
| 3B190 | During the illness that led to death, did (s)he have fast  breathing? | YES |  | | ➡ |  | |  |  |  |
|  |  | NO |  | |  | 3B210 | |  |  |  |
|  |  | Don’t know |  | | ➡ | 3B210 | |  |  |  |
|  |  | Refuse to answer |  | | ➡ | 3B210 | |  |  |  |
| 3B200 | For how many days did the fast breathing last? | DAYS |  | |  |  | |  |  |  |
| 3B210 | Did (s)he have breathlessness? | YES |  | | ➡ |  | |  |  |  |
|  |  | NO |  | |  | 3B242 | |  |  |  |
|  |  | Don’t know |  | | ➡ | 3B242 | |  |  |  |
|  |  | Refuse to answer |  | | ➡ | 3B242 | |  |  |  |
| 3B220 | For how many weeks did (s)he have breathlessness? | WEEKS |  |  |  | |  |  | |  |
| 3B242 | During the illness that led to death, did (s)he have  difficulty breathing? | YES |  | | ➡ |  | |  |  |  |
|  |  | NO |  | |  | 3B250 | |  |  |  |
|  |  | Don’t know |  | | ➡ | 3B250 | |  |  |  |
|  |  | Refuse to answer |  | | ➡ | 3B250 | |  |  |  |
| 3B244 | For how many days did the difficulty breathing last? | DAYS |  | |  |  | |  |  |  |
| 3B250 | Did you see the lower chest wall/ribs being pulled in as  the child breathed? | YES |  | |  |  | |  |  |  |
|  |  | NO |  | |  |  | |  |  |  |
|  |  | Don’t know |  | |  |  | |  |  |  |
|  |  | Refuse to answer |  | |  |  | |  |  |  |
| 3B260 | During the illness that led to death did his/her breathing  sound like any of the following: | Stridor |  | |  |  | |  |  |  |
|  |  | Grunting |  | |  |  | |  |  |  |
|  |  | Wheezing |  | |  |  | |  |  |  |
|  |  | NO |  | |  |  | |  |  |  |
|  |  | Don’t know |  | |  |  | |  |  |  |
|  |  | Refuse to answer |  | |  |  | |  |  |  |
| 3B280 | Did (s)he have diarrhoea? | YES |  | | ➡ |  | |  |  |  |
|  |  | NO |  | |  | 3B310 | |  |  |  |
|  |  | Don’t know |  | | ➡ | 3B310 | |  |  |  |
|  |  | Refuse to answer |  | | ➡ | 3B310 | |  |  |  |
| 3B300 | At any time during the final illness was there blood in  the stools? | YES |  | |  |  | |  |  |  |
|  |  | NO |  | |  |  | |  |  |  |
|  |  | Don’t know |  | |  |  | |  |  |  |
|  |  | Refuse to answer |  | |  |  | |  |  |  |
| 3B310 | Did (s)he vomit? | YES |  | | ➡ |  | |  |  |  |
|  |  | NO |  | |  | 3B330 | |  |  |  |
|  |  | Don’t know |  | | ➡ | 3B330 | |  |  |  |
|  |  | Refuse to answer |  | | ➡ | 3B330 | |  |  |  |
| 3B315 | For how many days before death did (s)he vomit? | DAYS |  | |  |  | |  |  |  |
| 3B320 | Did (s)he vomit blood? | YES |  | |  |  | |  |  |  |
|  |  | NO |  | |  |  | |  |  |  |
|  |  | Don’t know |  | |  |  | |  |  |  |
|  |  | Refuse to answer |  | |  |  | |  |  |  |

| 3B330 | Did (s)he have any abdominal problem? | YES |  |  |  |
| --- | --- | --- | --- | --- | --- |
|  |  | NO |  |  |  |
|  |  | Don’t know |  |  |  |
|  |  | Refuse to answer |  |  |  |
| 3B360 | Did (s)he have a more than usually  protruding abdomen? | YES |  |  |  |
|  |  | NO |  |  |  |
|  |  | Don’t know |  |  |  |
|  |  | Refuse to answer |  |  |  |
| 3B440 | Was (s)he unconscious for more than 24  hours before death? | YES |  |  |  |
|  |  | NO |  |  |  |
|  |  | Don’t know |  |  |  |
|  |  | Refuse to answer |  |  |  |
| 3B460 | Did (s)he have convulsions? | YES |  |  |  |
|  |  | NO |  |  |  |
|  |  | Don’t know |  |  |  |
|  |  | Refuse to answer |  |  |  |
| 3B530 | Did (s)he have any skin problems? | YES |  |  |  |
|  |  | NO |  |  |  |
|  |  | Don’t know |  |  |  |
|  |  | Refuse to answer |  |  |  |
| 3B560 | During the illness that led to death, did (s)he have any  skin rash? | YES |  |  |  |
|  |  | NO |  |  |  |
|  |  | Don’t know |  |  |  |
|  |  | Refuse to answer |  |  |  |
| 3B594 | During the illness that led to death, did he/she have  areas of the skin that turned black? | YES |  |  |  |
|  |  | NO |  |  |  |
|  |  | Don’t know |  |  |  |
|  |  | Refuse to answer |  |  |  |
| 3B596 | During the illness that led to death, did (s)he bleed  from anywhere? | YES |  |  |  |
|  |  | NO |  |  |  |
|  |  | Don’t know |  |  |  |
|  |  | Refuse to answer |  |  |  |
| 3B750 | Did (s)he have yellow discoloration of the eyes? | YES |  |  |  |
|  |  | NO |  |  |  |
|  |  | Don’t know |  |  |  |
|  |  | Refuse to answer |  |  |  |



| **SECTION 5. NEONATAL AND CHILD HISTORY, SIGNS AND SYMPTOMS** | | | | | | | | | | | |  |
| --- | --- | --- | --- | --- | --- | --- | --- | --- | --- | --- | --- | --- |
| 3D070 | How old was the baby when the fatal illness started? | DAYS | |  |  | |  | | |  | |  |
|  |  |  |  |  |  |  |  | | |  | |  |
|  |  |  | |  |  | |  | | |  | |  |
| 3D100 | Was the child part of a multiple birth? | YES | | |  | | ➡ | | |  | |  |
|  |  | NO | | |  | |  |  |  | 3D104 | |  |
|  |  | Don’t know | | |  | | ➡ | | | 3D104 | |  |
|  |  | Refuse to answer | | |  | | ➡ | | | 3D104 | |  |
| 3D102 | Was the child the first, second, or later in the birth  order? | First | | |  | |  | | |  | |  |
|  |  | Second or later | | |  | |  | | |  | |  |
| 3D104 | Is the mother still alive? | YES | | |  | | ➡ | | | 3D155 | |  |
|  |  | NO | | |  | | ➡ | | |  | |  |
|  |  | Don’t know | | |  | |  |  |  | 3D155 | |  |
|  |  | Refuse to answer | | |  | | ➡ | | | 3D155 | |  |
| 3D106 | Did the mother die during or after the delivery? | During delivery | | |  | | ➡ 3D155 | | | | |  |
|  |  | After delivery | | |  | |  |  |  |  |  |  |
|  |  |  |  |  |  |  |  |  |  |  |  |  |
| 3D108a | How many months after the delivery did the mother  die? | MONTHS | |  |  | |  | | |  | |  |
| 3D108b | How many days after the delivery did the mother die? | DAYS | |  |  | |  | | |  | |  |
| 3D155 | Where was the deceased born? | Hospital | |  |  | |  | | |  | |  |
|  |  |  |  |  |  |  |  | | |  | |  |
|  |  | Other health facility | | |  | |  | | |  | |  |
|  |  | Home | | |  | |  | | |  | |  |
|  |  | On route to hospital or facility | | |  | |  |  |  |  |  |  |
|  |  | Other | | |  | |  | | |  | |  |
|  |  | Don’t know | | |  | |  | | |  | |  |
|  |  | Refuse to answer | | |  | |  | | |  | |  |
| 3D165 | Did the mother receive professional assistance during the delivery? | YES | | |  | |  | | |  | |  |
|  |  | NO | | |  | |  | | |  | |  |
|  |  | Don’t know | | |  | |  | | |  | |  |
|  |  | Refuse to answer | | |  | |  | | |  | |  |
| 3D180 | At birth, was the baby of usual size? | YES | | |  | |  | | |  | |  |
|  |  | NO | | |  | |  | | |  | |  |
|  |  | Don’t know | | |  | |  | | |  | |  |
|  |  | Refuse to answer | | |  | |  | | |  | |  |
| 3D190 | At birth, was the baby smaller than usual,  (weighing less than 2.5 kg)? | YES | | |  | | ➡ | | | 3D201 | |  |
|  |  | NO | | |  | |  | | |  | |  |
|  |  | Don’t know | | |  | |  | | |  | |  |
|  |  | Refuse to answer | | |  | |  | | |  | |  |
| 3D200 | At birth, was the baby larger than usual, weighing over 4.5 kg)? | YES | | |  | |  | | |  | |  |
|  |  | NO | | |  | |  | | |  | |  |
|  |  | Don’t know | | |  | |  | | |  | |  |
|  |  | Refuse to answer | | |  | |  | | |  | |  |
| 3D201 | What was the weight (in grams) of the deceased at birth? | GRAMS |  | | | |  | | |  | |  |
|  |  |  |  |  |  |  |  | | |  | |  |
|  |  |  |  | |  | |  | | |  | |  |
| 3D210 | How many months long was the pregnancy before the  child was born? | MONTHS |  | |  | |  | | |  | |  |
|  |  |  |  | |  |  |  | | |  | |  |
|  |  |  |  | |  | |  | | |  | |  |
| 3D215 | Were there any complications in the late part of the pregnancy (defined as the last 3 months, before labour)? | YES | | |  | |  | | |  | |  |
|  |  | NO | | |  | |  | | |  | |  |
|  |  | Don’t know | | |  | |  | | |  | |  |
|  |  | Refuse to answer | | |  | |  | | |  | |  |
| 3D221 | Were there any complications during labour or delivery? | YES | | |  | |  | | |  | |  |
|  |  | NO | | |  | |  | | |  | |  |
|  |  | Don’t know | | |  | |  | | |  | |  |
|  |  | Refuse to answer | | |  | |  | | |  | |  |
| 3D230 | Was any part of the baby physically abnormal at time of delivery? (for example: body part too large or too small, additional growth on body)? | YES | | |  | |  | | |  | |  |
|  |  | NO | | |  | |  | | |  | |  |
|  |  | Don’t know | | |  | |  | | |  | |  |
|  |  | Refuse to answer | | |  | |  | | |  | |  |
| 3D240 | Did the baby/ child have a swelling or defect on the back? | YES | | |  | |  |  |  | |  |  |
|  |  | NO | | |  | |  |  |  | |  |  |
|  |  | Don’t know | | |  | |  |  |  | |  |  |
|  |  | Refuse to answer | | |  | |  |  |  | |  |  |
| 3D241 | Did the baby/ child have a very large head? | YES | | |  | |  | ➡ | | | 3D251 |  |
|  |  | NO | | |  | |  |  |  | |  |  |
|  |  | Don’t know | | |  | |  |  |  | |  |  |
|  |  | Refuse to answer | | |  | |  |  |  | |  |  |
| 3D242 | Did the baby/ child have a very small head? | YES | | |  | |  |  |  | |  |  |
|  |  | NO | | |  | |  |  |  | |  |  |
|  |  | Don’t know | | |  | |  |  |  | |  |  |
|  |  | Refuse to answer | | |  | |  |  |  | |  |  |
| 3D251 | Did the baby stop moving in the womb before labour started? | YES | | |  | |  | ➡ | | |  |  |
|  |  | NO | | |  | |  |  |  |  | 3D255 |  |
|  |  | Don’t know | | |  | |  | ➡ | | | 3D255 |  |
|  |  | Refuse to answer | | |  | |  | ➡ | | | 3D255 |  |
| 3D251a | How many days before labour did you or the mother last feel the baby move? (maybe the respondent or health worker had examined the mother) |  | | |  | |  |  |  | |  |  |
|  |  |  | | |  | |  |  |  | |  |  |
|  |  | DAYS | | |  |  |  |  |  | |  |  |
|  |  |  | | |  | |  |  |  | |  |  |
| 3D251b | How many hours before labour did you or the mother last feel the baby move? (maybe the respondent or health worker had examined the mother) |  | | |  | |  |  |  | |  |  |
|  |  |  | | |  | |  |  |  | |  |  |
|  |  | HOURS | | |  |  |  |  |  | |  |  |
|  |  |  | | |  | |  |  |  | |  |  |
| 3D253 | Was the baby born 24 hours or more after  the water broke? | YES | | |  | |  | | | | |  |
|  |  | NO | | |  | |  |  |  |  |  |  |
|  |  | Don’t know | | |  | |  |  |  |  |  |  |
|  |  | Refuse to answer | | |  | |  |  |  |  |  |  |
| 3D254 | Was the liquor foul smelling? | YES | | |  | |  |  |  | |  |  |
|  |  | NO | | |  | |  |  |  | |  |  |
|  |  | Don’t know | | |  | |  |  |  | |  |  |
|  |  | Refuse to answer | | |  | |  |  |  | |  |  |
| **How was the baby delivered?** | | | | | | | | | | | |  |
| 3D258 | Was the delivery normal vaginal, without forceps or vacuum? | YES | | |  | ➡ | | | | | 3D261 |  |
|  |  | NO | | |  |  | | | | |  |  |
|  |  | Don’t know | | |  |  | | | | |  |  |
|  |  | Refuse to answer | | |  |  | | | | |  |  |
| 3D259 | Was the delivery vaginal, with forceps or vacuum? | YES | | |  | ➡ | | | | | 3D261 |  |
|  |  | NO | | |  |  | | | | |  |  |
|  |  | Don’t know | | |  |  | | | | |  |  |
|  |  | Refuse to answer | | |  |  | | | | | |  |
| 3D260 | Was the delivery a caesarean section? | YES | | |  |  | | | | | |  |
|  |  | NO | | |  |  | | | | |  |  |
|  |  | Don’t know | | |  |  | | | | |  |  |
|  |  | Refuse to answer | | |  |  | | | | | |  |
| 3D261 | Did you/the mother receive any vaccinations since reaching adulthood including during this pregnancy? | YES | | |  | ➡ | | | | |  |  |
|  |  | NO | | |  |  |  |  |  |  | 3D267 |  |
|  |  | Don’t know | | |  | ➡ | | | | | 3D267 |  |
|  |  | Refuse to answer | | |  | ➡ | | | | | 3D267 |  |
| 3D263 | How many doses? | DOSES | | |  |  | | | | |  |  |
| 3D265 | Did the mother receive tetanus toxoid (TT) vaccine? | YES | | |  |  | | | | |  |  |
|  |  | NO | | |  |  | | | | | |  |
|  |  | Don’t know | | |  |  | | | | | |  |
|  |  | Refuse to answer | | |  |  | | | | |  |  |
| 3D267 | How many births, including stillbirths, did the baby's mother have before this baby? | BIRTHS | | |  |  | | | | |  |  |
|  |  |  |  |  |  |  | | | | |  |  |
|  | | | | | | | | | | | |  |



| 3D269 | During the last 3 months of pregnancy, labour or delivery, did the baby's mother suffer from high blood pressure? | YES |  |  |  |  |
| --- | --- | --- | --- | --- | --- | --- |
|  |  | NO |  |  |  |  |
|  |  | Don’t know |  |  |  |  |
|  |  | Refuse to answer |  |  |  |  |
| 3D271 | Did the baby's mother have foul smelling vaginal  discharge during pregnancy or after delivery? | YES |  |  |  |  |
|  |  | NO |  |  |  |  |
|  |  | Don’t know |  |  |  |  |
|  |  | Refuse to answer |  |  |  |  |
| 3D273 | During the last 3 months of pregnancy, labour or delivery, did the baby's mother suffer from convulsions? | YES |  |  |  |  |
|  |  | NO |  |  |  |  |
|  |  | Don’t know |  |  |  |  |
|  |  | Refuse to answer |  |  |  |  |
| 3D275 | During the last 3 months of pregnancy did the baby's  mother suffer from blurred vision? | YES |  |  |  |  |
|  |  | NO |  |  |  |  |
|  |  | Don’t know |  |  |  |  |
|  |  | Refuse to answer |  |  |  |  |
| 3D276 | Did the baby's mother have vaginal bleeding during the  last 3 months of pregnancy but before labour started? | YES |  |  |  |  |
|  |  | NO |  |  |  |  |
|  |  | Don’t know |  |  |  |  |
|  |  | Refuse to answer |  |  |  |  |
| 3D277 | Did the baby's bottom, feet, arm or hand come out of the vagina before its head? | YES |  |  |  |  |
|  |  | NO |  |  |  |  |
|  |  | Don’t know |  |  |  |  |
|  |  | Refuse to answer |  |  |  |  |
| 3D278 | Was the umbilical cord wrapped more than once around the neck of the child at birth? | YES |  |  |  |  |
|  |  | NO |  |  |  |  |
|  |  | Don’t know |  |  |  |  |
|  |  | Refuse to answer |  |  |  |  |
| 3D280 | Was the baby blue in colour at birth? | YES |  |  |  |  |
|  |  | NO |  |  |  |  |
|  |  | Don’t know |  |  |  |  |
|  |  | Refuse to answer |  |  |  |  |
| 3D285 | Did the baby ever cry? | YES |  | ➡ |  |  |
|  |  | NO |  |  | 3D298 |  |
|  |  | Don’t know |  | ➡ |  |  |
|  |  | Refuse to answer |  |  | 3D298 |  |
| 3D290 | Did the baby cry immediately after birth, even if only a little bit? | YES |  | ➡ |  |  |
|  |  | NO |  |  | 3D294 |  |
|  |  | Don’t know |  | ➡ | 3D294 |  |
|  |  | Refuse to answer |  | ➡ | 3D294 |  |
| 3D292 | How many minutes after birth did the baby first cry? | MINUTES |  |  |  |  |
| 3D294 | Did the baby stop being able to cry? | YES |  | ➡ |  |  |
|  |  | NO |  |  | 3D298 |  |
|  |  | Don’t know |  | ➡ | 3D298 |  |
|  |  | Refuse to answer |  | ➡ | 3D298 |  |
| 3D296 | How many hours before death did the baby stop crying? | HOURS |  |  |  |  |
|  |  |  |  |  |  |  |
|  |  |  |  |  |  |  |
| 3D298 | Did the baby ever move? | YES |  |  |  |  |
|  |  | NO |  |  |  |  |
|  |  | Don’t know |  |  |  |  |
|  |  | Refuse to answer |  |  |  |  |
| 3D299 | Did the baby ever breathe? | YES |  | ➡ |  |  |
|  |  | NO |  |  | 3D310 |  |
|  |  | Don’t know |  |  |  |  |
|  |  | Refuse to answer |  |  |  |  |
| 3D300 | Did the baby breathe immediately after birth, even a little? | YES |  |  |  |  |
|  |  | NO |  |  |  |  |
|  |  | Don’t know |  |  |  |  |
|  |  | Refuse to answer |  |  |  |  |

| 3D310 | Was the baby given assistance to breathe at birth? | YES |  |  |  |  |
| --- | --- | --- | --- | --- | --- | --- |
|  |  | NO |  |  |  |  |
|  |  | Don’t know |  |  |  |  |
|  |  | Refuse to answer |  |  |  |  |
| 3D320 | If the baby didn't show any sign of life, was it born dead?  Ask this question only if you answer three  questions(3D285, 3D298, 3D299) as ‘NO’ | YES |  | ➡ |  |  |
|  |  | NO |  |  | 3D345 |  |
|  |  | Don’t know |  | ➡ | 3D345 |  |
|  |  | Refuse to answer |  | ➡ | 3D345 |  |
|  |  |  |  |  |  |  |
| 3D325 | Were there any bruises or signs of injury on child's body after the birth?  Ask this question only if the baby was born dead | YES |  |  |  |  |
|  |  | NO |  |  |  |  |
|  |  | Don’t know |  |  |  |  |
|  |  | Refuse to answer |  |  |  |  |
| 3D330 | Was the dead baby macerated, that is, showed signs of decay?  Ask this question only if the baby was born dead | YES |  | ➡ | 3E100 |  |
|  |  |  |  |  |  |  |
|  |  | NO |  | ➡ | 3E100 |  |
|  |  |  |  |  |  |  |
|  |  | Don’t know |  | ➡ | 3E100 |  |
|  |  | Refuse to answer |  | ➡ | 3E100 |  |
| 3D345 | Did the baby stop suckling? | YES |  | ➡ |  |  |
|  |  | NO |  |  | 3D360 |  |
|  |  | Don’t know |  | ➡ | 3D360 |  |
|  |  | Refuse to answer |  | ➡ | 3D360 |  |
| 3D350 | How many days after birth did the baby stop suckling? | DAYS |  |  |  |  |
| 3D360 | Did the baby have convulsions starting within the first 24 hours of life? | YES |  | ➡ | 3D380 |  |
|  |  | NO |  |  |  |  |
|  |  | Don’t know |  |  |  |  |
|  |  | Refuse to answer |  |  |  |  |
| 3D370 | Did the baby have convulsions starting more than 24  hours after birth? | YES |  |  |  |  |
|  |  | NO |  |  |  |  |
|  |  | Don’t know |  |  |  |  |
|  |  | Refuse to answer |  |  |  |  |
| 3D380 | Did the baby's body become stiff, with the back arched backwards? | YES |  |  |  |  |
|  |  | NO |  |  |  |  |
|  |  | Don’t know |  |  |  |  |
|  |  | Refuse to answer |  |  |  |  |
| 3D390 | During the illness that led to death, did the baby have a bulging or raised fontanelle? | YES |  | ➡ | 3D410 |  |
|  |  | NO |  |  |  |  |
|  |  | Don’t know |  |  |  |  |
|  |  | Refuse to answer |  |  |  |  |
| 3D400 | During the illness that led to death, did the baby have a sunken fontanelle? | YES |  |  |  |  |
|  |  | NO |  |  |  |  |
|  |  | Don’t know |  |  |  |  |
|  |  | Refuse to answer |  |  |  |  |
| 3D410 | Did the baby become unresponsive or unconscious soon after birth, within less than 24 hours? | YES |  | ➡ | 3D430 |  |
|  |  | NO |  |  |  |  |
|  |  | Don’t know |  |  |  |  |
|  |  | Refuse to answer |  |  |  |  |
| 3D420 | Did the baby become unresponsive or unconscious more than 24 hours after birth? | YES |  |  |  |  |
|  |  | NO |  |  |  |  |
|  |  | Don’t know |  |  |  |  |
|  |  | Refuse to answer |  |  |  |  |
| 3D430 | During the illness that led to death, did the baby become cold to touch? | YES |  |  |  |  |
|  |  | NO |  |  |  |  |
|  |  | Don’t know |  |  |  |  |
|  |  | Refuse to answer |  |  |  |  |
| 3D435 | During the illness that led to death, did the baby become lethargic, after a period of normal activity? | YES |  |  | |  |
|  |  | NO |  |  |  |  |
|  |  | Don’t know |  |  |  |  |
|  |  | Refuse to answer |  |  |  |  |

| 3D440 | Did the baby have redness or discharge from the umbilical cord stump? | YES |  |  |
| --- | --- | --- | --- | --- |
|  |  | NO |  |  |
|  |  | Don’t know |  |  |
|  |  | Refuse to answer |  |  |
| 3D445 | During the illness that led to death, did the baby have skin ulcer(s) or pits? | YES |  |  |
|  |  | NO |  |  |
|  |  | Don’t know |  |  |
|  |  | Refuse to answer |  |  |
| 3D450 | During the illness that led to death, did the baby have yellow skin, palms (hand) or soles (foot)? | YES |  |  |
|  |  | NO |  |  |
|  |  | Don’t know |  |  |
|  |  | Refuse to answer |  |  |
| 3D455 | Did the baby or infant appear to be healthy and then just die suddenly? | YES |  |  |
|  |  | NO |  |  |
|  |  | Don’t know |  |  |
|  |  | Refuse to answer |  |  |

**SECTION 6. HISTORY OF INJURIES/ACCIDENTS**

| 3E100 | Did (s)he suffer from any injury or accident that led to her/his death? | | YES |  |  |  |  | |
| --- | --- | --- | --- | --- | --- | --- | --- | --- |
|  |  |  | NO |  | ➡ | 3G110 |  | |
|  |  |  |  |  |  |  |  | |
|  |  |  | Don’t know |  | ➡ | 3G110 |  | |
|  |  |  | Refuse to answer |  | ➡ | 3G110 |  | |
| 3E102 | Was the injury intentionally inflicted by someone else? | | YES |  |  |  |  | |
|  |  |  | NO |  | ➡ | 3E115 |  | |
|  |  |  | Don’t know |  | ➡ | 3E115 |  | |
|  |  |  | Refuse to answer |  | ➡ | 3E115 |  | |
| 3E104 | Was (s)he injured by a firearm? | | YES |  |  |  |  | |
|  |  |  | NO |  |  |  |  | |
|  |  |  | Don’t know |  |  |  |  | |
|  |  |  | Refuse to answer |  |  |  |  | |
| 3E106 | Was (s)he stabbed, cut or pierced? | | YES |  |  |  |  | |
|  |  |  | NO |  |  |  |  | |
|  |  |  | Don’t know |  |  |  |  | |
|  |  |  | Refuse to answer |  |  |  |  | |
| 3E108 | Was (s)he strangled? | | YES |  |  |  |  | |
|  |  |  | NO |  |  |  |  | |
|  |  |  | Don’t know |  |  |  |  | |
|  |  |  | Refuse to answer |  |  |  |  | |
| 3E111 | Was (s)he injured by a blunt force? | | YES |  |  |  |  | |
|  |  |  | NO |  |  |  |  | |
|  |  |  | Don’t know |  |  |  |  | |
|  |  |  | Refuse to answer |  |  |  |  | |
| 3E112 | Was (s)he injured by burns? | | YES |  |  |  |  | |
|  |  |  | NO |  |  |  |  | |
|  |  |  | Don’t know |  |  |  |  | |
|  |  |  | Refuse to answer |  |  |  |  | |
| 3E115 | Was it a road traffic accident? | | YES |  | ➡ |  |  | |
|  |  |  | NO |  |  | 3E310 |  | |
|  |  |  | Don’t know |  | ➡ | 3E310 |  | |
|  |  |  | Refuse to answer |  | ➡ | 3E310 |  | |
| 3E120 | What was her/his role in the road traffic accident? | | Pedestrian |  |  |  |  | |
|  |  |  | In car or light vehicle |  |  |  |  | |
|  |  |  | In bus or heavy  vehicle |  |  |  |  | |
|  |  |  | On a motorcycle |  |  |  |  | |
|  |  |  | On a pedal cycle |  |  |  |  | |
|  |  |  | Other |  |  |  |  | |
| 3E170 | What was the counterpart that was hit during the road traffic accident? | | Pedestrian |  |  |  |  | |
|  |  |  | Stationary object |  |  |  |  | |
|  |  |  | Car or light vehicle |  |  |  |  | |
|  |  |  | Bus or heavy  vehicle |  |  |  |  | |
|  |  |  |  |  |  |  |  | |
|  |  |  | Motorcycle |  |  |  |  | |
|  |  |  | Pedal cycle |  |  |  |  | |
|  |  |  | Other |  |  |  |  | |
| 3E310 | Was (s)he injured in a fall? | | YES |  |  |  |  | |
|  |  |  | NO |  |  |  |  | |
|  |  |  | Don’t know |  |  |  |  | |
|  |  |  | Refuse to answer |  |  |  |  | |
| 3E320 | Did (s)he die of drowning? | | YES |  |  |  |  | |
|  |  |  | NO |  |  |  |  | |
|  |  |  | Don’t know |  |  |  |  | |
|  |  |  | Refuse to answer |  |  |  |  | |
| 3E330 | Did (s)he suffer from accidental burns? | YES | |  |  | | |  |
|  |  | NO | |  |  |  |  |  |
|  |  | Don’t know | |  |  |  |  |  |
|  |  | Refuse to answer | |  |  |  |  |  |
| 3E335 | Was (s)he accidentally injured by a blunt force? | YES | |  |  |  | |  |
|  |  | NO | |  |  |  | |  |
|  |  | Don’t know | |  |  |  | |  |
|  |  | Refuse to answer | |  |  |  | |  |
| 3E340 | Was (s)he accidentally injured by a plant/animal/insect that led to her/his death? | YES | |  | ➡ |  | |  |
|  |  | NO | |  |  | 3E500 | |  |
|  |  | Don’t know | |  | ➡ | 3E500 | |  |
|  |  | Refuse to answer | |  | ➡ | 3E500 | |  |
| 3E400 | What was the plant/animal/insect? | Dog | |  |  |  | |  |
|  |  | Snake | |  |  |  | |  |
|  |  | Insect or scorpion | |  |  |  | |  |
|  |  | Other | |  |  |  | |  |
|  |  | Don’t know | |  |  |  | |  |
| 3E500 | Was (s)he injured by a force of nature? | YES | |  |  |  | |  |
|  |  | NO | |  |  |  | |  |
|  |  | Don’t know | |  |  |  | |  |
|  |  | Refuse to answer | |  |  |  | |  |
| 3E510 | Was there any poisoning? | YES | |  |  |  | |  |
|  |  | NO | |  |  |  | |  |
|  |  | Don’t know | |  |  |  | |  |
|  |  | Refuse to answer | |  |  |  | |  |
| 3E520 | Was (s)he subject to violence/assault? | YES | |  |  |  | |  |
|  |  | NO | |  |  |  | |  |
|  |  | Don’t know | |  |  |  | |  |
|  |  | Refuse to answer | |  |  |  | |  |
| 3E530 | Was it electrocution? | YES | |  |  |  | |  |
|  |  | NO | |  |  |  | |  |
|  |  | Don’t know | |  |  |  | |  |
|  |  | Refuse to answer | |  |  |  | |  |

| **SECTION 7. HEALTH SERVICE UTILISATION** | | | | | |  |
| --- | --- | --- | --- | --- | --- | --- |
| 3G110 | Did (s)he receive any treatment for the illness that led to death? | YES |  |  |  |  |
|  |  | NO |  | ➡ | 3H100 |  |
|  |  | Don’t know |  | ➡ | 3H100 |  |
|  |  | Refuse to answer |  | ➡ | 3H100 |  |
| 3G120 | Did (s)he receive oral rehydration salts? | YES |  |  |  |  |
|  |  | NO |  |  |  |  |
|  |  | Don’t know |  |  |  |  |
|  |  | Refuse to answer |  |  |  |  |
| 3G130 | Did (s)he receive (or need) intravenous fluids (drip) treatment? | YES |  |  |  |  |
|  |  | NO |  |  |  |  |
|  |  | Don’t know |  |  |  |  |
|  |  | Refuse to answer |  |  |  |  |
| 3G140 | Did (s)he receive (or need) a blood transfusion? | YES |  |  |  |  |
|  |  | NO |  |  |  |  |
|  |  | Don’t know |  |  |  |  |
|  |  | Refuse to answer |  |  |  |  |
| 3G150 | Did (s)he receive (or need) treatment/food through a tube passed through the nose? | YES |  |  |  |  |
|  |  | NO |  |  |  |  |
|  |  | Don’t know |  |  |  |  |
|  |  | Refuse to answer |  |  |  |  |
| 3G160 | Did (s)he receive (or need) injectable antibiotics? | YES |  |  |  |  |
|  |  | NO |  |  |  |  |
|  |  | Don’t know |  |  |  |  |
|  |  | Refuse to answer |  |  |  |  |
| 3G165 | Did (s)he receive (or need) antiretroviral therapy (ART)? | YES |  |  |  |  |
|  |  | NO |  |  |  |  |
|  |  | Don’t know |  |  |  |  |
|  |  | Refuse to answer |  |  |  |  |
| 3G170 | Did (s)he receive (or need) an operation for the illness? | YES |  |  |  |  |
|  |  | NO |  |  |  |  |
|  |  | Don’t know |  |  |  |  |
|  |  | Refuse to answer |  |  |  |  |
| 3G190 | Was (s)he discharge from hospital very ill? | YES |  |  |  |  |
|  |  | NO |  |  |  |  |
|  |  | Don’t know |  |  |  |  |
|  |  | Refuse to answer |  |  |  |  |
| 3H100 | Has (s)he received immunization? | YES |  | ➡ |  |  |
|  |  | NO |  |  | 3H130 |  |
|  |  | Don’t know |  | ➡ | 3H130 |  |
|  |  | Refuse to answer |  | ➡ | 3H130 |  |
| 3H110 | Do you have the child's vaccination card? | YES |  | ➡ |  |  |
|  |  | NO |  |  | 3H130 |  |
|  |  | Don’t know |  | ➡ | 3H130 |  |
|  |  | Refuse to answer |  | ➡ | 3H130 |  |
| 3H120 | Can I see the vaccination card (note the vaccines the child received)? | YES |  |  |  |  |
|  |  | NO |  |  |  |  |
|  |  | Don’t know |  |  |  |  |
|  |  | Refuse to answer |  |  |  |  |
| 3H125 | Note vaccines here: |  |  |  |  |  |
|  | _____________________________________________________________________________ | | |  |  |  |
|  | _____________________________________________________________________________ | | |  |  |  |
|  | _____________________________________________________________________________ | | |  |  |  |
|  | _____________________________________________________________________________ | | |  |  |  |
|  |  |  |  |  |  |  |
| 3H130 | Was care sought outside the home while (s)he had this illness? | YES |  | ➡ |  |  |
|  |  | NO |  |  | 3H160 |  |
|  |  | Don’t know |  | ➡ | 3H160 |  |
|  |  | Refuse to answer |  | ➡ | 3H160 |  |

| 3H140 | Where or from whom did you seek care? | Traditional healer | |  |  |  |  |
| --- | --- | --- | --- | --- | --- | --- | --- |
|  |  | Homeopath | |  |  |  |  |
|  |  | Religious leader | |  |  |  |  |
|  |  | Private hospital | |  |  |  |  |
|  |  | Government hospital | |  |  |  |  |
|  |  | Government health  centre or clinic |  |  |  |  |  |
|  |  |  |  |  |  |  |  |
|  |  | Community-based  practitioner associated  with Health system | |  |  |  |  |
|  |  | Trained birth attendant | |  |  |  |  |
|  |  | Private physician | |  |  |  |  |
|  |  | Pharmacy | |  |  |  |  |
|  |  | Don’t know | |  |  |  |  |
|  |  | Refuse to answer | |  |  |  |  |
| 3H150 | Record the name and address of any hospital, health centre or clinic where care was sought __________________________________________________________________________ | | | |  |  |  |
|  |  |  |  |  |  |  |  |
|  | ___________________________________________________________ | |  |  |  |  |  |
|  |  |  |  |  |  |  |  |
| 3H160 | Did a health care worker tell you the cause of death? | YES | |  | ➡ |  |  |
|  |  | NO | |  |  | 3H180 |  |
|  |  | Don’t know | |  | ➡ | 3H180 |  |
|  |  | Refuse to answer | |  | ➡ | 3H180 |  |
| 3H170 | What did the health care worker say? | |  |  |  |  |  |
|  | ____________________________________________________________ | |  |  |  |  |  |
|  | _____________________________________________________________ | |  |  |  |  |  |
|  |  |  |  |  |  |  |  |
| 3H180 | Do you have any health records that belonged to the deceased? | YES | |  | ➡ |  |  |
|  |  | NO | |  |  | 3H330 |  |
|  |  | Don’t know | |  | ➡ | 3H330 |  |
|  |  | Refuse to answer | |  | ➡ | 3H330 |  |
| 3H190 | Can I see the health records? | YES | |  | ➡ |  |  |
|  |  | NO | |  |  | 3H330 |  |
|  |  | Don’t know | |  | ➡ | 3H330 |  |
|  |  | Refuse to answer | |  | ➡ | 3H330 |  |
| 3H200 | Record the date of the most recent (last) visit | DAY |  |  |  |  |  |
|  |  | MONTH |  |  |  |  |  |
|  |  | YEAR |  |  |  |  |  |
| 3H210 | Record the date of the last but one (second last) visit | DAY |  |  |  |  |  |
|  |  | MONTH |  |  |  |  |  |
|  |  | YEAR |  |  |  |  |  |
| 3H220 | Record the date of the last note on the health records | DAY |  |  |  |  |  |
|  |  | MONTH |  |  |  |  |  |
|  |  | YEAR |  |  |  |  |  |
| 3H230 | Record the weight (in kilogrammes) written at the most  recent (last) visit | [KG] | . | |  |  |  |
|  |  |  |  |  |  |  |  |
| 3H240 | Record the weight (in kilogrammes) written at the last but one (second last) visit | [KG] | . | |  |  |  |
|  |  |  |  |  |  |  |  |
| 3H250 | Transcribe the last note on the health records | |  |  |  |  |  |
|  | ____________________________________________________________________ | |  |  |  |  |  |
|  | ____________________________________________________________________ | |  |  |  |  |  |
|  |  |  |  |  |  |  |  |

| 3H330 | Has the deceased’s (biological) mother ever been tested for HIV? | YES |  | ➡ |  |  |
| --- | --- | --- | --- | --- | --- | --- |
|  |  | NO |  |  | 3H350 |  |
|  |  | Don’t know |  | ➡ | 3H350 |  |
|  |  | Refuse to answer |  | ➡ | 3H350 |  |
| 3H340 | Was the HIV test ever positive? | YES |  |  |  |  |
|  |  | NO |  |  |  |  |
|  |  | Don’t know |  |  |  |  |
|  |  | Refuse to answer |  |  |  |  |
| 3H350 | Has the deceased’s (biological) mother ever been told she had HIV/AIDS by a health worker? | YES |  |  |  |  |
|  |  | NO |  |  |  |  |
|  |  | Don’t know |  |  |  |  |
|  |  | Refuse to answer |  |  |  |  |
| **SECTION 8. BACKGROUND AND CONTEXT** | |  |  |  |  |  |
| 4A100 | In the final days before death, did s/he travel to a hospital or health facility? | YES |  | ➡ |  |  |
|  |  | NO |  |  | 4A150 |  |
|  |  | Don’t know |  | ➡ | 4A150 |  |
|  |  | Refuse to answer |  | ➡ | 4A150 |  |
| 4A110 | Did (s)he use motorised transport to get to the hospital or health facility? | YES |  |  |  |  |
|  |  | NO |  |  |  |  |
|  |  | Don’t know |  |  |  |  |
|  |  | Refuse to answer |  |  |  |  |
| 4A120 | Were there any problems during admission to the hospital or health facility? | YES |  |  |  |  |
|  |  | NO |  |  |  |  |
|  |  | Don’t know |  |  |  |  |
|  |  | Refuse to answer |  |  |  |  |
| 4A130 | Were there any problems with the way (s)he was treated (medical treatment, procedures, interpersonal attitudes, respect, dignity) in the hospital or health facility? | YES |  |  |  |  |
|  |  | NO |  |  |  |  |
|  |  | Don’t know |  |  |  |  |
|  |  | Refuse to answer |  |  |  |  |
| 4A140 | Were there any problems getting medications, or diagnostic tests in the hospital or health facility? | YES |  |  |  |  |
|  |  | NO |  |  |  |  |
|  |  | Don’t know |  |  |  |  |
|  |  | Refuse to answer |  |  |  |  |
| 4A150 | Does it take more than 2 hours to get to the nearest hospital or health facility from the deceased's household? | YES |  |  |  |  |
|  |  | NO |  |  |  |  |
|  |  |  |  |  |  |  |
|  |  | Don’t know |  |  |  |  |
|  |  | Refuse to answer |  |  |  |  |
| 4A160 | In the final days before death, were there any doubts about whether medical care was needed? | YES |  |  |  |  |
|  |  | NO |  |  |  |  |
|  |  |  |  |  |  |  |
|  |  |  |  |  |  |  |
|  |  | Don’t know |  |  |  |  |
|  |  | Refuse to answer |  |  |  |  |
| 4A170 | In the final days before death, was traditional medicine used? | YES |  |  |  |  |
|  |  | NO |  |  |  |  |
|  |  | Don’t know |  |  |  |  |
|  |  | Refuse to answer |  |  |  |  |
| 4A180 | In the final days before death, did anyone use a telephone or cell phone to call for help? | YES |  |  |  |  |
|  |  | NO |  |  |  |  |
|  |  | Don’t know |  |  |  |  |
|  |  | Refuse to answer |  |  |  |  |
| 4A190 | Over the course of illness, did the total costs of care and treatment prohibit other household payments? | YES |  |  |  |  |
|  |  | NO |  |  |  |  |
|  |  | Don’t know |  |  |  |  |
|  |  | Refuse to answer |  |  |  |  |

**SECTION 9. OPTIONAL OPEN NARRATIVE**

| 5A100 | Narrative Description |
| --- | --- |
|  | _________________________________________________________________________ |
|  |  |
|  | _________________________________________________________________________ |
|  | _________________________________________________________________________ |
|  | _________________________________________________________________________ |
|  | _________________________________________________________________________ |
|  | _________________________________________________________________________ |
|  |  |

**SECTION 10. DEATH CERTIFICATE**

| 6H260 | Was a death certificate issued? | YES |  | ➡ |  |  |
| --- | --- | --- | --- | --- | --- | --- |
|  |  | NO |  |  | End |  |
|  |  | Don’t know |  | ➡ | End |  |
|  |  | Refuse to answer |  | ➡ | End |  |
| 6H270 | Can I see the death certificate? | YES |  | ➡ |  |  |
|  |  | NO |  |  | End |  |
|  |  | Don’t know |  | ➡ | End |  |
|  |  | Refuse to answer |  | ➡ | End |  |
| 6H280 | Record the immediate cause of death from the certificate (line 1a) *  ____________________________________________________________ | |  | Duration 1(a) | |  |
|  |  |  |  |  |  |  |
| 6H290 | Record the first antecedent cause of death from the certificate (line 1b) ____________________________________________________________ | |  | Duration 1(b) | |  |
|  |  |  |  |  |  |  |
| 6H300 | Record the second antecedent cause of death from the certificate (line 1c)  ____________________________________________________________ | |  | Duration 1(c) | |  |
|  |  |  |  |  |  |  |
| 6H310 | Record the third antecedent cause of death from the certificate (line 1d) ____________________________________________________________ | |  | Duration 1(d) | |  |
|  |  |  |  |  |  |  |
| 6H320 | Record the contributing cause(s) of death from the certificate (part 2) __________________________________________________________ | |  |  |  |  |
|  |  |  |  |  |  |  |

**VERBAL AUTOPSY**

**QUESTIONNAIRE (II)**

**Death of an infant aged 1-11 months**

| **NO.** | **QUESTIONS AND FILTERS** |  | | **ANSWER** | |  |  | **SKIP** | |  |
| --- | --- | --- | --- | --- | --- | --- | --- | --- | --- | --- |
| 0A100a | Is this a region of high HIV/AIDS prevalence? | YES | | | |  |  |  |  |  |
|  |  | NO | | | |  |  |  |  |  |
| 0A100b | Is this a region of high malaria prevalence? | YES | | | |  |  |  |  |  |
|  |  | NO | | | |  |  |  |  |  |
| **SECTION 1. INFORMATION ON THE DECEASED INFANT** | |  | |  | |  |  |  |  |  |
| 1A100a | What was the first or given name(s) of the deceased? _________________________________________________________ | | | | |  |  |  |  |  |
|  |  |  |  |  |  |  |  |  |  |  |
| 1A100b | What was the **surname (or family name)** of the deceased?  _________________________________________________________ | | | | | |  |  |  |  |
|  |  |  |  |  |  |  |  |  |  |  |
| 1A110 | What was the sex of the deceased? |  | | MALE | |  |  | ➡ 1A200 | |  |
|  |  |  | |  |  |  |  |  |  |  |
|  |  | FEMALE | | | |  |  |  |  |  |
| 1A400 | Was this a woman who died more than 42 days but less than 1 year after being pregnant or delivering a baby? | YES | | | |  |  |  | |  |
|  |  | NO | | | |  |  |  |  |  |
|  |  | Don’t know | | | |  |  |  |  |  |
|  |  | Refuse to answer | | | |  |  |  |  |  |
| 1A401 | Was this a woman who died more than 42days after being pregnant or delivering a baby? | YES | | | |  |  |  | |  |
|  |  | NO | | | |  |  |  |  |  |
|  |  | Don’t know | | | |  |  |  |  |  |
|  |  | Refuse to answer | | | |  |  |  |  |  |
| 1A200 | Is the date of birth known? | YES | | | |  |  | ➡ |  |  |
|  |  | NO | | | |  |  |  | 1A220 |  |
|  |  | Refuse to answer | | | |  |  | ➡ | 1A220 |  |
| 1A210 | When was the deceased born? |  | | DAY | |  |  |  |  |  |
|  |  |  | |  |  |  |  |  |  |  |
|  |  |  | | MONTH | |  |  |  |  |  |
|  |  |  | | YEAR | |  |  |  |  |  |
|  |  |  | |  | |  |  |  |  |  |
| 1A220 | Is the date of death known? | YES | | | |  |  | ➡ |  |  |
|  |  | NO | | | |  |  |  | AAAA |  |
|  |  | Refuse to answer | | | |  |  | ➡ | AAAA |  |
| 1A230 | When did (s)he die? |  | | DAY | |  |  |  |  |  |
|  |  |  | | MONTH | |  |  |  |  |  |
|  |  |  | | YEAR | |  |  |  |  |  |
|  |  |  | |  | |  |  |  |  |  |
| AAAA | Put infant's age in months |  | | MONTHS | |  |  |  |  |  |
|  |  |  | |  |  |  |  |  |  |  |
|  |  |  | |  | |  |  |  |  |  |
| 1A500 | What was her/his citizenship/nationality? | Citizen at birth | | | |  |  |  |  |  |
|  |  | Naturalized citizen | | | |  |  |  |  |  |
|  |  |  | | Foreign national | |  |  |  |  |  |
|  |  | Don’t know | | | |  |  |  |  |  |
| 1A510 | What was her/his ethnicity? _________________________________________ | | | | | |  |  |  |  |
|  |  |  |  |  |  |  |  |  |  |  |
| 1A520 | What was her/his place of birth? ___________________________________ | | | |  | |  |  |  |  |
|  |  |  |  |  |  | |  |  |  |  |
| 1A530 | What was her/his place of usual residence? (The place where the person lived most of the year) ________________ | | | | | |  |  |  |  |
|  |  |  |  |  |  |  |  |  |  |  |
| 1A540 | What was her/his place of normal residence 1 to 5 years before death? ____________ | | | | | |  |  |  |  |
| 1A550 | Where did death occur? (specify country, province, district, village) _______________ | | | | | |  |  |  |  |
|  |  |  |  |  |  |  |  |  |  |  |
| 1A560 | Where did the deceased die? | | Hospital | | |  |  |  |  |  |
|  |  |  | Other health facility | | |  |  |  |  |  |
|  |  |  | Home | | |  |  |  |  |  |
|  |  |  | On route to facility or hospital | | |  |  |  |  |  |
|  |  |  | Other | | |  |  |  |  |  |
|  |  |  | Don’t know | | |  |  |  |  |  |
|  |  |  | Refuse to answer | | |  |  |  |  |  |

| **SECTION 2. Vital Registration and Certification** | | | | | | | | | | | | | | | | | | | | |  |  |  |
| --- | --- | --- | --- | --- | --- | --- | --- | --- | --- | --- | --- | --- | --- | --- | --- | --- | --- | --- | --- | --- | --- | --- | --- |
| 1A700 | | Death registration number/ certificate __________________________________________________________ | | | | | | | | | | | |  | | | | | | |  | |  |
|  | |  |  |  |  |  |  |  |  |  |  |  |  |  | | | | | | |  | |  |
| 1A710 | | Date of registration | | DAY | | | | |  | | | | |  | | | | | | |  | |  |
|  | |  |  |  |  |  |  |  |  |  |  |  |  |  | | | | | | |  | |  |
|  | |  |  | MONTH | | | | |  | | | | |  | | | | | | |  | |  |
|  | |  |  | YEAR | | | | |  | | | | |  | | | | | | |  | |  |
|  | |  | |  | | | | |  | | | | |  | | | | | | |  | |  |
| 1A720 | | Place of registration  _____________________________________________________________ | | | | | | | | | | | |  | | | | | | |  | |  |
|  | |  |  |  |  |  |  |  |  |  |  |  |  |  | | | | | | |  | |  |
| 1A730 | | National identification number of deceased _____________________________________________________________ | | | | | | | | | | | |  | | | | | | |  | |  |
|  | |  |  |  |  |  |  |  |  |  |  |  |  |  | | | | | | |  | |  |
| **SECTION 3. Information on the respondent and background about interview** | | | | | | | | | | | | | | | | | | | | |  | |  |
|  | |  | |  | | | | |  | | | | |  | | | | | | |  | |  |
| 2A100 | | What is the name of VA respondent? | |  | | | | |  | | | | |  | | | | | | |  | |  |
|  | | ____________________________________________ | | | | | | | | | | | |  | | | | | | |  | |  |
|  | |  | |  | | | | |  | | | | |  | | | | | | |  | |  |
| 2A110 | | What is the respondent's relationship to the deceased? | | Parent | | | | |  | | | | |  | | | | | | |  | |  |
|  | |  |  | Child | | | | |  | | | | |  | | | | | | |  | |  |
|  | |  |  | Other family member | | | | |  | | | | |  | | | | | | |  | |  |
|  | |  |  | Friend | | | | |  | | | | |  | | | | | | |  | |  |
|  | |  |  | Health worker | | | | |  | | | | |  | | | | | | |  | |  |
|  | |  |  | Public official | | | | |  | | | | |  | | | | | | |  | |  |
|  | |  |  | Another relationship | | | | |  | | | | |  | | | | | | |  | |  |
| 2A115 | | Did the respondent live with the deceased in the period leading to her/his death? | | YES | | | | |  | | | | |  | | | | | | |  | |  |
|  | |  |  | NO | | | | |  | | | | |  | | | | | | |  | |  |
|  | |  |  | Don’t know | | | | |  | | | | |  | | | | | | |  | |  |
|  | |  |  | Refuse to answer | | | | |  | | | | |  | | | | | | |  | |  |
| 2A120 | | Name of VA interviewer  _______________________________________________ | | | | | | | | | | | |  | | | | | | |  | |  |
| 2A130 | | Time at start of interview | | hh:mm 24h | | | | ___፡___ | | | | | |  | | | | | | |  | |  |
|  | |  | |  |  |  |  |  |  |  |  |  |  |  | | | | | | |  | |  |
| 2A135 | | Time at end of interview | | hh:mm 24h | | | | ___፡___ | | | | | |  | | | | | | |  | |  |
|  | |  | |  |  |  |  |  |  |  |  |  |  |  | | | | | | |  | |  |
| 2A140 | | Date of interview | | DAY | | | |  | | | | | |  | | | | | | |  | |  |
|  | |  |  | MONTH | | | |  | | | | | |  | | | | | | |  | |  |
|  | |  |  | YEAR | | | |  | | | | | |  | | | | | | |  | |  |
| 2A150 | | Did the respondent give consent? | | YES | | | |  | | | | | |  | | | | | | |  | |  |
|  | |  |  | NO | | | |  | | | | | |  | | | | | | |  | |  |
| 3A280 | | During which season did (s)he die? | | WET | | | |  | | | | | |  | | | | | | |  |  |  |
|  | |  |  | DRY | | | |  | | | | | |  | | | | | | |  |  |  |
| 3A300 | | For how many days was (s)he ill before (s)he died? | DAYS | | | |  | | | | | | | |  |  | | | | |  | |  |
| 3A310 | | Did (s)he die suddenly? | | YES | | | |  | | | | | |  | | | | | | |  |  |  |
|  | |  |  | NO | | | |  | | | | | |  | | | | | | |  |  |  |
|  | |  |  | Don’t know | | | |  | | | | | |  | | | | | | |  |  |  |
|  | |  |  | Refuse to answer | | | |  | | | | | |  | | | | | | |  |  |  |
| 3A3100 | | What age of the respondent (in full years) |  | | | | |  | | | |  | | | | | | | | |  |  |  |
| 3A3101 | | What was her/his marital status? | Single | | | | |  | | | |  |  | | | | |  | | |  |  |  |
|  |  |  | Married | | | | |  | | | |  |  | | | | |  | | |  |  |  |
|  | |  | Life partner | | | | |  | | | |  |  | | | | |  | | |  |  |  |
|  | |  | Divorced | | | | |  | | | |  |  | | | | |  | | |  |  |  |
|  | |  | Widowed | | | | |  | | | |  |  | | | | |  | | |  |  |  |
|  | |  | Too young to be  married | | | | |  | | | |  |  | | | | |  | | |  |  |  |
|  | |  | Don’t know | | | | |  | | | |  |  | | | | |  | | |  |  |  |
|  | |  | Refuse to answer | | | | |  | | | |  |  | | | | |  | | |  |  |  |
| 3A 3101 | | What was the date of marriage? |  | | | DAY | |  | | | |  |  | | | | |  | | |  |  |  |
|  | |  |  | | |  |  |  |  |  |  |  |  | | | | |  | | |  |  |  |
|  | |  |  | | | MONTH | |  | | | |  |  | | | | |  | | |  |  |  |
|  | |  |  | | | YEAR | |  | | | |  |  | | | | |  | | |  |  |  |
| 3A3102 | | What was her/his highest level of schooling? | No formal education | | | | |  | | | |  |  | | | | |  | | |  |  |  |
|  | |  | Primary school(1-8 Grade) | | | | |  | | | |  |  | | | | |  | | |  |  |  |
|  | |  | Secondary school (9-12 Grade) | | | | |  | | | |  |  | | | | |  | | |  |  |  |
|  | |  | Higher then secondary school | | | | |  | | | |  |  | | | | |  | | |  |  |  |
|  | |  | Don’t know | | | | |  | | | |  |  | | | | |  | | |  |  |  |
|  | |  | Refuse to answer | | | | |  | | | |  |  | | | | |  | | |  |  |  |
| 3A3103 | | Was (s)he able to read and write? (select 'yes' also if only one of either reading or writing is know to the respondent) | YES | | | | |  | | |  | | | | | | | | | |  |  |  |
|  | |  | NO | | | | |  | | |  | | | | | | | | | |  |  |  |
|  | |  | Don’t know | | | | |  | | |  | | | | | | | | | |  |  |  |
|  | |  | Refuse to answer | | | | |  | | |  | | | | | | | | | |  |  |  |
| 3A3104 | | What was her/his economic activity status in year prior to death? | Mainly  unemployed | | | | |  | | |  | |  | | | | |  | | |  |  |  |
|  | |  | Mainly employed | | | | |  | | |  | |  | | | | |  | | |  |  |  |
|  | |  | Home-maker | | | | |  | | |  | |  | | | | |  | | |  |  |  |
|  | |  | Pensioner | | | | |  | | |  | |  | | | | |  | | |  |  |  |
|  | |  | Student | | | | |  | | |  | |  | | | | |  | | |  |  |  |
|  | |  | Other | | | | |  | | |  | |  | | | | |  | | |  |  |  |
|  | |  | Don’t know | | | | |  | | |  | |  | | | | |  | | |  |  |  |
|  |  |  | Refuse to answer | | | | |  | | |  | |  | | | | |  | | |  |  |  |
| 3A3105 | | What was her/his occupation, that is, what kind of work does (s)he mainly do?  ________________________________________________ | | | | | | | | |  | | | | | | | | | |  |  |  |
|  | |  |  |  |  |  |  |  |  |  |  |  |  |  |  |  |  |  |  |  |  |  |  |
|  | |  |  |  |  |  |  |  |  |  |  |  |  |  |  |  |  |  |  |  |  |  |  |
| **SECTION 4: MEDICAL HISTORY ASSOCIATED WITH FINAL ILLNESS.** | | | | | | | | | | | | | | | | | | |  |  |  |  |  |
| 3A100 | Was there any diagnosis by a physician or health worker of tuberculosis? | | | | YES | | | | |  | | |  | | | |  | |  | | | | |
|  |  |  |  |  | NO | | | | |  | | |  |  |  |  |  | |  | | | | |
|  |  |  |  |  | Don’t know | | | | |  | | |  | | | |  | |  | | | | |
|  |  |  |  |  | Refuse to answer | | | | |  | | |  | | | |  | |  | | | | |
| 3A110 | Was there any diagnosis by a physician or health worker of HIV/AIDS? | | | | YES | | | | |  | | |  | | | |  | |  | | | | |
|  |  |  |  |  | NO | | | | |  | | |  | | | |  | |  | | | | |
|  |  |  |  |  | Don’t know | | | | |  | | |  | | | |  | |  | | | | |
|  |  |  |  |  | Refuse to answer | | | | |  | | |  | | | |  | |  | | | | |
| 3A120 | Did (s)he have a recent positive test by a physician or health worker for malaria? | | | | YES | | | | |  | | |  | | | |  | |  | | | | |
|  |  |  |  |  | NO | | | | |  | | |  | | | |  | |  | | | | |
|  |  |  |  |  | Don’t know | | | | |  | | |  | | | |  | |  | | | | |
|  |  |  |  |  | Refuse to answer | | | | |  | | |  | | | |  | |  | | | | |
| 3A130 | Did (s)he have a recent negative test by a physician or health worker for malaria? | | | | YES | | | | |  | | |  | | | |  | |  | | | | |
|  |  |  |  |  | NO | | | | |  | | |  |  |  |  |  | |  | | | | |
|  |  |  |  |  | Don’t know | | | | |  | | |  | | | |  | |  | | | | |
|  |  |  |  |  | Refuse to answer | | | | |  | | |  | | | |  | |  | | | | |
| 3A135 | Was there any diagnosis by a physician or health worker of dengue fever? | | | | YES | | | | |  | | |  | | | |  | |  | | | | |
|  |  |  |  |  | NO | | | | |  | | |  |  |  |  |  | |  | | | | |
|  |  |  |  |  | Don’t know | | | | |  | | |  | | | |  | |  | | | | |
|  |  |  |  |  | Refuse to answer | | | | |  | | |  | | | |  | |  | | | | |
| 3A140 | Was there any diagnosis by a physician or health worker of measles? | | | | YES | | | | |  | | |  | | | |  | |  | | | | |
|  |  |  |  |  | NO | | | | |  | | |  |  |  |  |  | |  | | | | |
|  |  |  |  |  | Don’t know | | | | |  | | |  | | | |  | |  | | | | |
|  |  |  |  |  | Refuse to answer | | | | |  | | |  | | | |  | |  | | | | |
| 3A150 | Was there any diagnosis by a physician or health worker of high blood pressure? | | | | YES | | | | |  | | |  | | | |  | |  | | | | |
|  |  |  |  |  | NO | | | | |  | | |  | | | |  | |  | | | | |
|  |  |  |  |  | Don’t know | | | | |  | | |  | | | |  | |  | | | | |
|  |  |  |  |  | Refuse to answer | | | | |  | | |  | | | |  | |  | | | | |
| 3A160 | Was there any diagnosis by a physician or health worker of heart disease? | | | | YES | | | | |  | | |  | | | |  | |  | | | | |
|  |  |  |  |  | NO | | | | |  | | |  |  |  |  |  | |  | | | | |
|  |  |  |  |  | Don’t know | | | | |  | | |  | | | |  | |  | | | | |
|  |  |  |  |  | Refuse to answer | | | | |  | | |  | | | |  | |  | | | | |
| 3A170 | Was there any diagnosis by a physician or health worker of diabetes? | | | | YES | | | | |  | | |  | | | |  | |  | | | | |
|  |  |  |  |  | NO | | | | |  | | |  | | | |  | |  | | | | |
|  |  |  |  |  | Don’t know | | | | |  | | |  | | | |  | |  | | | | |
|  |  |  |  |  | Refuse to answer | | | | |  | | |  | | | |  | |  | | | | |
| 3A180 | Was there any diagnosis by a physician or health worker of asthma? | | | | YES | | | | |  | | |  | | | |  | |  | | | | |
|  |  |  |  |  | NO | | | | |  | | |  |  |  |  |  | |  | | | | |
|  |  |  |  |  | Don’t know | | | | |  | | |  | | | |  | |  | | | | |
|  |  |  |  |  | Refuse to answer | | | | |  | | |  | | | |  | |  | | | | |
| 3A190 | Was there any diagnosis by a physician or health worker of epilepsy? | | | | YES | | | | |  | | |  | | | |  | |  | | | | |
|  |  |  |  |  | NO | | | | |  | | |  | | | |  | |  | | | | |
|  |  |  |  |  | Don’t know | | | | |  | | |  | | | |  | |  | | | | |
|  |  |  |  |  | Refuse to answer | | | | |  | | |  | | | |  | |  | | | | |
| 3A200 | Was there any diagnosis by a physician or health worker of cancer? | | | | YES | | | | |  | | |  | | | |  | |  | | | | |
|  |  |  |  |  | NO | | | | |  | | |  |  |  |  |  | |  | | | | |
|  |  |  |  |  | Don’t know | | | | |  | | |  | | | |  | |  | | | | |
|  |  |  |  |  | Refuse to answer | | | | |  | | |  | | | |  | |  | | | | |
| 3A210 | Was there any diagnosis by a physician or health worker of Chronic Obstructive Pulmonary Disease (COPD)? | | | | YES | | | | |  | | |  | | | |  | |  | | | | |
|  |  |  |  |  | NO | | | | |  | | |  | | | |  | |  | | | | |
|  |  |  |  |  | Don’t know | | | | |  | | |  | | | |  | |  | | | | |
|  |  |  |  |  | Refuse to answer | | | | |  | | |  | | | |  | |  | | | | |
| 3A220 | Was there any diagnosis by a physician or health worker of dementia? | | | | YES | | | | |  | | |  | | | | | |  |  |  |  |  |
|  |  |  |  |  | NO | | | | |  | | |  | | | | | |  |  |  |  |  |
|  |  |  |  |  | Don’t know | | | | |  | | |  | | | | | |  |  |  |  |  |
|  |  |  |  |  | Refuse to answer | | | | |  | | |  | | | | | |  |  |  |  |  |
| 3A230 | Was there any diagnosis by a physician or health worker of depression? | | | | YES | | | | |  | | |  | | | | | |  |  |  |  |  |
|  |  |  |  |  | NO | | | | |  | | |  | | | | | |  |  |  |  |  |
|  |  |  |  |  | Don’t know | | | | |  | | |  | | | | | |  |  |  |  |  |
|  |  |  |  |  | Refuse to answer | | | | |  | | |  | | | | | |  |  |  |  |  |
| 3A240 | Was there any diagnosis by a physician or health worker of stroke? | | | | YES | | | | |  | | |  | | | | | |  |  |  |  |  |
|  |  |  |  |  | NO | | | | |  | | |  | | | | | |  |  |  |  |  |
|  |  |  |  |  | Don’t know | | | | |  | | |  | | | | | |  |  |  |  |  |
|  |  |  |  |  | Refuse to answer | | | | |  | | |  | | | | | |  |  |  |  |  |
| 3A250 | Was there any diagnosis by a physician or health worker of sickle cell disease? | | | | YES | | | | |  | | |  | | | | | |  |  |  |  |  |
|  |  |  |  |  | NO | | | | |  | | |  | | | | | |  |  |  |  |  |
|  |  |  |  |  | Don’t know | | | | |  | | |  | | | | | |  |  |  |  |  |
|  |  |  |  |  | Refuse to answer | | | | |  | | |  | | | | | |  |  |  |  |  |
| 3A260 | Was there any diagnosis by a physician or health worker of kidney disease? | | | | YES | | | | |  | | |  | | | | | |  |  |  |  |  |
|  |  |  |  |  | NO | | | | |  | | |  | | | | | |  |  |  |  |  |
|  |  |  |  |  | Don’t know | | | | |  | | |  | | | | | |  |  |  |  |  |
|  |  |  |  |  | Refuse to answer | | | | |  | | |  | | | | | |  |  |  |  |  |
| 3A270 | Was there any diagnosis by a physician or health worker of liver disease? | | | | YES | | | | |  | | |  | | | | | |  |  |  |  |  |
|  |  |  |  |  | NO | | | | |  | | |  | | | | | |  |  |  |  |  |
|  |  |  |  |  | Don’t know | | | | |  | | |  | | | | | |  |  |  |  |  |
|  |  |  |  |  | Refuse to answer | | | | |  | | |  | | | | | |  |  |  |  |  |

| **SECTION 5: GENERAL SIGNS AND SYMPTOMS ASSOCIATED WITH FINAL ILLNESS** | | | | | | | | | | | |  |  |  |  |  |
| --- | --- | --- | --- | --- | --- | --- | --- | --- | --- | --- | --- | --- | --- | --- | --- | --- |
| 3B100 | Did (s)he have a fever? | YES | |  | | ➡ | | |  | | |  | | |  |  |
|  |  | NO | |  | |  |  |  | 3B130 | | |  | | |  |  |
|  |  | Don’t know | |  | | ➡ | | | 3B130 | | |  | | |  |  |
|  |  | Refuse to answer | |  | | ➡ | | | 3B130 | | |  | | |  |  |
| 3B110 | How many days did the fever last? | DAYS | |  | |  | | |  | | |  | | |  |  |
| 3B115 | How severe was the fever? | Mild | |  | |  | | |  | | |  | | |  |  |
|  |  | Moderate | |  | |  | | |  | | |  | | |  |  |
|  |  | Severe | |  | |  | | |  | | |  | | |  |  |
| 3B120 | Did (s)he have night sweats? | YES | |  | |  | | |  | | |  | | |  |  |
|  |  | NO | |  | |  | | |  | | |  | | |  |  |
|  |  | Don’t know | |  | |  | | |  | | |  | | |  |  |
|  |  | Refuse to answer | |  | |  | | |  | | |  | | |  |  |
| 3B130 | Did (s)he have a cough? | YES | |  | |  | | |  | | |  | | |  |  |
|  |  | NO | |  | | ➡ | | | 3B180 | | |  | | |  |  |
|  |  | Don’t know | |  | | ➡ | | | 3B180 | | |  | | |  |  |
|  |  | Refuse to answer | |  | | ➡ | | | 3B180 | | |  | | |  |  |
| 3B140 | For how many days did (s)he have a cough? | DAYS | |  | |  | | |  | | |  | | |  |  |
| 3B150 | Was the cough productive, with sputum? | YES | |  | |  | | |  | | |  | | |  |  |
|  |  | NO | |  | |  | | |  | | |  | | |  |  |
|  |  | Don’t know | |  | |  | | |  | | |  | | |  |  |
|  |  | Refuse to answer | |  | |  | | |  | | |  | | |  |  |
| 3B155 | Was the cough very severe? | YES | |  | |  | | |  | | |  | | |  |  |
|  |  | NO | |  | |  | | |  | | |  | | |  |  |
|  |  | Don’t know | |  | |  | | |  | | |  | | |  |  |
|  |  | Refuse to answer | |  | |  | | |  | | |  | | |  |  |
| 3B160 | Did (s)he cough up blood? | YES | |  | |  | | |  | | |  | | |  |  |
|  |  | NO | |  | |  | | |  | | |  | | |  |  |
|  |  | Don’t know | |  | |  | | |  | | |  | | |  |  |
|  |  | Refuse to answer | |  | |  | | |  | | |  | | |  |  |
| 3B180 | Did (s)he have any breathing problem? | YES | |  | |  | | |  | | |  | | |  |  |
|  |  | NO | |  | |  | | |  | | |  | | |  |  |
|  |  | Don’t know | |  | |  | | |  | | |  | | |  |  |
|  |  | Refuse to answer | |  | |  | | |  | | |  | | |  |  |
| 3B190 | During the illness that led to death, did (s)he have fast breathing? | YES | |  | | ➡ | | |  | | |  | | |  |  |
|  |  | NO | |  | |  |  |  | 3B210 | | |  | | |  |  |
|  |  | Don’t know | |  | | ➡ | | | 3B210 | | |  | | |  |  |
|  |  | Refuse to answer | |  | | ➡ | | | 3B210 | | |  | | |  |  |
| 3B200 | For how many days did the fast breathing last? | DAYS | |  | |  | | |  | | |  | | |  |  |
| 3B210 | Did (s)he have breathlessness? | YES | |  | | ➡ | | |  | | |  | | |  |  |
|  |  | NO | |  | |  |  |  | 3B242 | | |  | | |  |  |
|  |  | Don’t know | |  | | ➡ | | | 3B242 | | |  | | |  |  |
|  |  | Refuse to answer | |  | | ➡ | | | 3B242 | | |  | | |  |  |
| 3B220 | For how many weeks did (s)he have breathlessness? | WEEKS | |  |  | | |  | |  |  | | | | | |
| 3B230 | Was (s)he unable to carry out daily routines due to breathlessness? | YES | |  | |  | | |  | | |  | | |  |  |
|  |  | NO | |  | |  |  |  |  | | |  | | |  |  |
|  |  | Don’t know | |  | |  | | |  | | |  | | |  |  |
|  |  | Refuse to answer | |  | |  | | |  | | |  | | |  |  |
| 3B240 | Was (s)he breathless while lying flat? | YES | |  | |  | | |  | | |  | | |  |  |
|  |  | NO | |  | |  |  |  |  | | |  | | |  |  |
|  |  | Don’t know | |  | |  | | |  | | |  | | |  |  |
|  |  | Refuse to answer | |  | |  | | |  | | |  | | |  |  |
| 3B242 | During the illness that led to death, did (s)he have difficulty breathing? | YES | |  | | ➡ | | |  | | |  | | |  |  |
|  |  | NO | |  | |  |  |  | 3B260 | | |  | | |  |  |
|  |  | Don’t know | |  | | ➡ | | | 3B260 | | |  | | |  |  |
|  |  | Refuse to answer | |  | | ➡ | | | 3B260 | | |  | | |  |  |
| 3B246 | Was the difficulty continuous or on and off? | Continuous | |  | |  | | |  | | |  | | |  |  |
|  |  | On and off | |  | |  |  |  |  |  |  |  | | |  |  |
| 3B260 | During the illness that led to death did his/her breathing sound like any of the following: Stridor, Grunting, Wheezing | Stridor | |  | |  | | |  | | |  | | |  |  |
|  |  | Grunting | |  | |  | | |  | | |  | | |  |  |
|  |  | Wheezing | |  | |  | | |  | | |  | | |  |  |
|  |  | NO | |  | |  | | |  | | |  | | |  |  |
|  |  | Don’t know | |  | |  | | |  | | |  | | |  |  |
|  |  | Refuse to answer | |  | |  | | |  | | |  | | |  |  |
| 3B270 | Did (s)he have severe chest pain? | YES | |  | | ➡ | | |  | | |  | | |  |  |
|  |  | NO | |  | |  |  |  | 3B280 | | |  | | |  |  |
|  |  | Don’t know | |  | | ➡ | | | 3B280 | | |  | | |  |  |
|  |  | Refuse to answer | |  | | ➡ | | | 3B280 | | |  | | |  |  |
| 3B272 | How many days before death did (s)he have severe chest pain? | DAYS | |  | |  | | |  | | |  | | |  |  |
| 3B274 | How many minutes did the pain last? | MINUTES | |  | |  | | |  | | |  | | |  |  |
| 3B280 | Did (s)he have diarrhoea? | YES | |  | | ➡ | | |  | | |  | | |  |  |
|  |  | NO | |  | |  |  |  | 3B310 | | |  | | |  |  |
|  |  | Don’t know | |  | | ➡ | | | 3B310 | | |  | | |  |  |
|  |  | Refuse to answer | |  | | ➡ | | | 3B310 | | |  | | |  |  |
| 3B290 | For how many days did (s)he have diarrhoea? | DAYS | |  | |  | | |  | | |  | | |  |  |
| 3B300 | At any time during the final illness was there blood in the stools? | YES | |  | |  | | |  | | |  | | |  |  |
|  |  | NO | |  | | ➡ | | | 3B310 | | |  | | |  |  |
|  |  | Don’t know | |  | | ➡ | | | 3B310 | | |  | | |  |  |
|  |  | Refuse to answer | |  | | ➡ | | | 3B310 | | |  | | |  |  |
| 3B305 | Was there blood in the stool up until death? | YES | |  | |  | | |  | | |  | | |  |  |
|  |  | NO | |  | |  | | |  | | |  | | |  |  |
|  |  | Don’t know | |  | |  | | |  | | |  | | |  |  |
|  |  | Refuse to answer | |  | |  | | |  | | |  | | |  |  |
| 3B310 | Did (s)he vomit? | YES | |  | | ➡ | | |  | | |  | | |  |  |
|  |  | NO | |  | |  |  |  | 3B330 | | |  | | |  |  |
|  |  | Don’t know | |  | | ➡ | | | 3B330 | | |  | | |  |  |
|  |  | Refuse to answer | |  | | ➡ | | | 3B330 | | |  | | |  |  |
| 3B315 | For how many days before death did (s)he vomit? | DAYS | |  | |  | | |  | | |  | | |  |  |
| 3B320 | Did (s)he vomit blood? | YES | |  | |  | | |  | | |  | | |  |  |
|  |  | NO | |  | |  | | |  | | |  | | |  |  |
|  |  | Don’t know | |  | |  | | |  | | |  | | |  |  |
|  |  | Refuse to answer | |  | |  | | |  | | |  | | |  |  |
| 3B325 | Was the vomit black? | YES | |  | |  | | |  | | |  | | |  |  |
|  |  | NO | |  | |  | | |  | | |  | | |  |  |
|  |  | Don’t know | |  | |  | | |  | | |  | | |  |  |
|  |  | Refuse to answer | |  | |  | | |  | | |  | | |  |  |
| 3B330 | Did (s)he have any abdominal problem? | YES | |  | |  | | | | | |  | |  |  |  |
|  |  | NO | |  | |  | | | | | |  | |  |  |  |
|  |  | Don’t know | |  | |  | | | | | |  | |  |  |  |
|  |  | Refuse to answer | |  | |  | | | | | |  | |  |  |  |
| 3B340 | Did (s)he have severe abdominal pain? | YES | |  | |  | | | | | |  | |  |  |  |
|  |  | NO | |  | | ➡ 3B360 | | | | | |  | |  |  |  |
|  |  | Don’t know | |  | | ➡ 3B360 | | | | | |  | |  |  |  |
|  |  | Refuse to answer | |  | | ➡ 3B360 | | | | | |  | |  |  |  |
| 3B350 | For how many days before death did (s)he have severe abdominal pain? | DAYS | |  | |  | | |  | | |  | | |  |  |
| 3B355 | Was the pain in the upper or lower abdomen? | Upper abdomen | |  | |  | | | | | |  | |  |  |  |
|  |  | Lower abdomen | |  | |  | | | | | |  | |  |  |  |
| 3B360 | Did (s)he have a more than usually protruding abdomen? | | YES |  | | |  | | | | | |  | | |  |
|  |  |  | NO |  | | | ➡ 3B380 | | | | | |  | | |  |
|  |  |  | Don’t know |  | | | ➡ 3B380 | | | | | |  | | |  |
|  |  |  | Refuse to answer |  | | | ➡ 3B380 | | | | | |  | | |  |
| 3B370 | For how many days did (s)he have a more than usually protruding abdomen? | DAYS | |  | |  | | |  | | |  | | |  |  |
| 3B375 | How rapidly did (s)he develop the protruding abdomen? | Rapidly | |  | |  | | | | | |  | | |  |  |
|  |  | Slowly | |  | |  | | | | | |  | | |  |  |
|  | |  | |  | |  | | | | | | | | |  |  |
| 3B380 | Did (s)he have any mass in the abdomen? | | YES |  | | |  | | | | | |  | | |  |
|  |  |  | NO |  | | | ➡ 3B400 | | | | | |  | | |  |
|  |  |  | Don’t know |  | | | ➡ 3B400 | | | | | |  | | |  |
|  |  |  | Refuse to answer |  | | | ➡ 3B400 | | | | | |  | | |  |
| 3B390 | For how many days before death did (s)he have a mass in the abdomen? | DAYS | |  | |  | | |  | | |  | | |  |  |
| 3B400 | Did (s)he have a severe headache? | | YES |  | | |  | | | | | |  | | |  |
|  |  |  | NO |  | | |  | | | | | |  | | |  |
|  |  |  | Don’t know |  | | |  | | | | | |  | | |  |
|  |  |  | Refuse to answer |  | | |  | | | | | |  | | |  |
| 3B405 | Did (s)he have a stiff neck during illness that led to death? | | YES |  | | |  | | | | | |  | | |  |
|  |  |  | NO |  | | | ➡ 3B409 | | | | | |  | | |  |
|  |  |  | Don’t know |  | | | ➡ 3B409 | | | | | |  | | |  |
|  |  |  | Refuse to answer |  | | | ➡ 3B409 | | | | | |  | | |  |
| 3B407 | For how many days before death did (s)he have stiff neck? | DAYS | |  | |  | | |  | | |  | | |  |  |
| 3B409 | Did (s)he have a painful neck during the illness that led to death? | | YES |  | | |  | | | | | |  | | |  |
|  |  |  | NO |  | | | ➡ 3B420 | | | | | |  | | |  |
|  |  |  | Don’t know |  | | | ➡ 3B420 | | | | | |  | | |  |
|  |  |  | Refuse to answer |  | | | ➡ 3B420 | | | | | |  | | |  |
| 3B410 | For how many days before death did (s)he have a painful neck? | DAYS | |  | |  | | |  | | |  | | |  |  |
| 3B420 | Did (s)he have mental confusion? | | YES |  | | |  | | | | | |  | | |  |
|  |  |  | NO |  | | | ➡ 3B440 | | | | | |  | | |  |
|  |  |  | Don’t know |  | | | ➡ 3B440 | | | | | |  | | |  |
|  |  |  | Refuse to answer |  | | | ➡ 3B440 | | | | | |  | | |  |
| 3B430 | For how many months did (s)he have mental confusion? | MONTHS | |  | |  | | |  | | |  | | |  |  |
| 3B440 | Was (s)he unconscious for more than 24 hours before death? | | YES |  | | |  | | | | | |  | | |  |
|  |  |  | NO |  | | | ➡ 3B460 | | | | | |  | | |  |
|  |  |  | Don’t know |  | | | ➡ 3B460 | | | | | |  | | |  |
|  |  |  | Refuse to answer |  | | | ➡ 3B460 | | | | | |  | | |  |
| 3B450 | Did the unconsciousness start suddenly, quickly (at least within a single day)? | | YES |  | | |  | | | | | |  | | |  |
|  |  |  | NO |  | | |  | | | | | |  | | |  |
|  |  |  | Don’t know |  | | |  | | | | | |  | | |  |
|  |  |  | Refuse to answer |  | | |  | | | | | |  | | |  |
| 3B455 | Did the unconsciousness continue until death? | | YES |  | | |  | | | | | |  | | |  |
|  |  |  | NO |  | | |  | | | | | |  | | |  |
|  |  |  | Don’t know |  | | |  | | | | | |  | | |  |
|  |  |  | Refuse to answer |  | | |  | | | | | |  | | |  |
| 3B460 | Did (s)he have convulsions? | | YES |  | | |  | | | | | |  | | |  |
|  |  |  | NO |  | | | ➡ 3B490 | | | | | |  | | |  |
|  |  |  | Don’t know |  | | | ➡ 3B490 | | | | | |  | | |  |
|  |  |  | Refuse to answer |  | | | ➡ 3B490 | | | | | |  | | |  |
| 3B465 | Did (s)he experience any generalized convulsions or fits during the illness that led to death? | | YES |  | | |  | | | | | |  | | |  |
|  |  |  | NO |  | | |  | | | | | |  | | |  |
|  |  |  | Don’t know |  | | |  | | | | | |  | | |  |
|  |  |  | Refuse to answer |  | | |  | | | | | |  | | |  |
| 3B470 | For how many minutes did the convulsions last? | MINUTES | |  | |  | | |  | | |  | | |  |  |
| 3B480 | Did (s)he become unconscious immediately after the convulsion? | | YES |  | | |  | | | | | |  | | |  |
|  |  |  | NO |  | | |  | | | | | |  | | |  |
|  |  |  | Don’t know |  | | |  | | | | | |  | | |  |
|  |  |  | Refuse to answer |  | | |  | | | | | |  | | |  |
| 3B490 | Did (s)he have any urine problems? | | YES |  | | |  | | | | | |  | | |  |
|  |  |  | NO |  | | |  | | | | | |  | | |  |
|  |  |  | Don’t know |  | | |  | | | | | |  | | |  |
|  |  |  | Refuse to answer |  | | |  | | | | | |  | | |  |
| 3B500 | Did (s)he pass no urine at all? | | YES |  | | |  | | | | | |  | | |  |
|  |  |  | NO |  | | |  | | | | | |  | | |  |
|  |  |  | Don’t know |  | | |  | | | | | |  | | |  |
|  |  |  | Refuse to answer |  | | |  | | | | | |  | | |  |
| 3B510 | Did (s)he go to urinate more often than usual? | | YES |  | | |  | | | | | |  | | |  |
|  |  |  | NO |  | | |  | | | | | |  | | |  |
|  |  |  | Don’t know |  | | |  | | | | | |  | | |  |
|  |  |  | Refuse to answer |  | | |  | | | | | |  | | |  |
| 3B520 | During the final illness did (s)he ever pass blood in the urine? | | YES |  | | |  | | | | | |  | | |  |
|  |  |  | NO |  | | |  | | | | | |  | | |  |
|  |  |  | Don’t know |  | | |  | | | | | |  | | |  |
|  |  |  | Refuse to answer |  | | |  | | | | | |  | | |  |
| 3B530 | Did (s)he have any skin problems? | | YES |  | | |  | | | | | |  | | |  |
|  |  |  | NO |  | | |  | | | | | |  | | |  |
|  |  |  | Don’t know |  | | |  | | | | | |  | | |  |
|  |  |  | Refuse to answer |  | | |  | | | | | |  | | |  |
| 3B535 | Did (s)he have sores? | | YES |  | | |  | | | | | |  | | |  |
|  |  |  | NO |  | | | ➡ 3B540 | | | | | |  | | |  |
|  |  |  | Don’t know |  | | | ➡ 3B540 | | | | | |  | | |  |
|  |  |  | Refuse to answer |  | | | ➡ 3B540 | | | | | |  | | |  |
| 3B537 | Did the sores have clear fluid or pus? | | YES |  | | |  | | | | | |  | | |  |
|  |  |  | NO |  | | |  | | | | | |  | | |  |
|  |  |  | Don’t know |  | | |  | | | | | |  | | |  |
|  |  |  | Refuse to answer |  | | |  | | | | | |  | | |  |
| 3B540 | Did (s)he have any ulcers, abscess or sores anywhere except on the feet? | | YES |  | | |  | | | | | |  | | |  |
|  |  |  | NO |  | | |  | | | | | |  | | |  |
|  |  |  | Don’t know |  | | |  | | | | | |  | | |  |
|  |  |  | Refuse to answer |  | | |  | | | | | |  | | |  |
| 3B542 | Did (s)he have an ulcer (pit) on the foot? | | YES |  | | |  | | | | | |  | | |  |
|  |  |  | NO |  | | | ➡ 3B560 | | | | | |  | | |  |
|  |  |  | Don’t know |  | | | ➡ 3B560 | | | | | |  | | |  |
|  |  |  | Refuse to answer |  | | | ➡ 3B560 | | | | | |  | | |  |
| 3B544 | Did the ulcer ooze pus? | | YES |  | | |  | | | | | |  | | |  |
|  |  |  | NO |  | | | ➡ 3B550 | | | | | |  | | |  |
|  |  |  | Don’t know |  | | | ➡ 3B550 | | | | | |  | | |  |
|  |  |  | Refuse to answer |  | | | ➡ 3B550 | | | | | |  | | |  |
| 3B546 | For how many days did the ulcer ooze pus? | DAYS | |  | |  | | |  | | |  | | |  |  |
| 3B550 | Did (s)he have any ulcers, abscess or sores on the feet that were not also on other parts of the body? | | YES |  | | |  | | | | | |  | | |  |
|  |  |  | NO |  | | |  | | | | | |  | | |  |
|  |  |  | Don’t know |  | | |  | | | | | |  | | |  |
|  |  |  | Refuse to answer |  | | |  | | | | | |  | | |  |
| 3B560 | During the illness that led to death, did (s)he have any skin rash? | | YES |  | | |  | | | | | |  | | |  |
|  |  |  | NO |  | | | ➡ 3B596 | | | | | |  | | |  |
|  |  |  | Don’t know |  | | | ➡ 3B596 | | | | | |  | | |  |
|  |  |  | Refuse to answer |  | | | ➡ 3B596 | | | | | |  | | |  |
| 3B570 | For how many days did (s)he have the skin rash? | DAYS | |  | |  | | |  | | |  | | |  |  |
| 3B575 | Where was the rash? | | Face |  | | |  | | | | | |  | | |  |
|  |  |  | Trunk or abdomen |  | | |  | | | | | |  | | |  |
|  |  |  | Extremities |  | | |  | | | | | |  | | |  |
|  |  |  | Everywhere |  | | |  | | | | | |  | | |  |
| 3B580 | Did (s)he have measles rash (use local term)? | | YES |  | | |  | | | | | |  | | |  |
|  |  |  | NO |  | | |  | | | | | |  | | |  |
|  |  |  | Don’t know |  | | |  | | | | | |  | | |  |
|  |  |  | Refuse to answer |  | | |  | | | | | |  | | |  |
| 3B590 | Did (s)he ever have shingles or herpes zoster? | | YES |  | | |  | | | | | |  | | |  |
|  |  |  | NO |  | | |  | | | | | |  | | |  |
|  |  |  | Don’t know |  | | |  | | | | | |  | | |  |
|  |  |  | Refuse to answer |  | | |  | | | | | |  | | |  |
| 3B596 | During the illness that led to death, did (s)he bleed from anywhere? | | YES |  | | |  | | | | | |  | | |  |
|  |  |  | NO |  | | | ➡ 3B610 | | | | | |  | | |  |
|  |  |  | Don’t know |  | | | ➡ 3B610 | | | | | |  | | |  |
|  |  |  | Refuse to answer |  | | | ➡ 3B610 | | | | | |  | | |  |

| 3B600 | | Did (s)he bleed from the nose, mouth or anus? | | | | YES | |  | | |  | | | |  | |
| --- | --- | --- | --- | --- | --- | --- | --- | --- | --- | --- | --- | --- | --- | --- | --- | --- |
|  |  |  |  |  |  | NO | |  | | |  |  |  |  |  | |
|  |  |  |  |  |  | Don’t know | |  | | |  |  |  |  |  | |
|  |  |  |  |  |  | Refuse to answer | |  | | |  |  |  |  |  | |
| 3B610 | | Did (s)he have noticeable weight loss? | | | | YES | |  | | |  | | | |  | |
|  | |  |  |  |  | NO | |  | | | ➡ 3B630 | | | |  | |
|  | |  |  |  |  | Don’t know | |  | | | ➡ 3B630 | | | |  | |
|  | |  |  |  |  | Refuse to answer | |  | | | ➡ 3B630 | | | |  | |
| 3B620 | | Was (s)he severely thin or wasted? | | | | YES | |  | | |  | | | |  | |
|  | |  |  |  |  | NO | |  | | |  | | | |  | |
|  | |  |  |  |  | Don’t know | |  | | |  | | | |  | |
|  | |  |  |  |  | Refuse to answer | |  | | |  | | | |  | |
| 3B630 | | During the illness that led to death, did s/he have a whitish rash inside the mouth or on the tongue? | | | | YES | |  | | |  | | | |  | |
|  | |  |  |  |  | NO | |  | | |  | | | |  | |
|  | |  |  |  |  | Don’t know | |  | | |  | | | |  | |
|  | |  |  |  |  | Refuse to answer | |  | | |  | | | |  | |
| 3B640 | | Did (s)he have stiffness of the whole body or was unable to open the mouth? | | | | YES | |  | | |  | | | |  | |
|  | |  |  |  |  | NO | |  | | |  | | | |  | |
|  | |  |  |  |  | Don’t know | |  | | |  | | | |  | |
|  | |  |  |  |  | Refuse to answer | |  | | |  | | | |  | |
| 3B650 | | Did (s)he have puffiness of the face? | | | | YES | |  | | |  | | | |  | |
|  | |  |  |  |  | NO | |  | | | ➡ 3B654 | | | |  | |
|  | |  |  |  |  | Don’t know | |  | | | ➡ 3B654 | | | |  | |
|  | |  |  |  |  | Refuse to answer | |  | | | ➡ 3B654 | | | |  | |
| 3B652 | | For how many days did (s)he have puffiness of the face? | | | DAYS | | |  | |  | | |  |  | |  |
| 3B654 | | During the illness that led to death, did (s)he have swelling in the armpits? | | | | YES | |  | | |  | | | |  | |
|  | |  |  |  |  | NO | |  | | |  | | | |  | |
|  | |  |  |  |  | Don’t know | |  | | |  | | | |  | |
|  | |  |  |  |  | Refuse to answer | |  | | |  | | | |  | |
| 3B656 | | During the illness that led to death, did (s)he have swollen legs or feet? | | | | YES | |  | | |  | | | |  | |
|  | |  |  |  |  | NO | |  | | | ➡ 3B660 | | | |  | |
|  | |  |  |  |  | Don’t know | |  | | | ➡ 3B660 | | | |  | |
|  | |  |  |  |  | Refuse to answer | |  | | | ➡ 3B660 | | | |  | |
| 3B658 | | How many days did the swelling last? | | | DAYS | | |  | |  | | |  |  | |  |
| 3B660 | | Did (s)he have both feet swollen? | | | | YES | |  | | |  | | | |  | |
|  | |  |  |  |  | NO | |  | | |  | | | |  | |
|  | |  |  |  |  | Don’t know | |  | | |  | | | |  | |
|  | |  |  |  |  | Refuse to answer | |  | | |  | | | |  | |
| 3B665 | | Did (s)he have general puffiness all over hi(s)her body? | | | | YES | |  | | |  | | | |  | |
|  | |  |  |  |  | NO | |  | | |  | | | |  | |
|  | |  |  |  |  | Don’t know | |  | | |  | | | |  | |
|  | |  |  |  |  | Refuse to answer | |  | | |  | | | |  | |
| 3B670 | | Did (s)he have any lumps? | | | | YES | |  | | |  | | | |  | |
|  | |  |  |  |  | NO | |  | | |  | | | |  | |
|  | |  |  |  |  | Don’t know | |  | | |  | | | |  | |
|  | |  |  |  |  | Refuse to answer | |  | | |  | | | |  | |
| 3B680 | | Did (s)he have any lumps or lesions in the mouth? | | | | YES | |  | | |  | | | |  | |
|  | |  |  |  |  | NO | |  | | |  | | | |  | |
|  | |  |  |  |  | Don’t know | |  | | |  | | | |  | |
|  | |  |  |  |  | Refuse to answer | |  | | |  | | | |  | |
| 3B690 | | Did (s)he have any lumps on the neck? | | | | YES | |  | | |  | | | |  | |
|  | |  |  |  |  | NO | |  | | |  | | | |  | |
|  | |  |  |  |  | Don’t know | |  | | |  | | | |  | |
|  | |  |  |  |  | Refuse to answer | |  | | |  | | | |  | |
| 3B700 | | Did (s)he have any lumps on the armpit? | | | | YES | |  | | |  | | | |  | |
|  | |  |  |  |  | NO | |  | | |  | | | |  | |
|  | |  |  |  |  | Don’t know | |  | | |  | | | |  | |
|  | |  |  |  |  | Refuse to answer | |  | | |  | | | |  | |
| 3B710 | | Did (s)he have any lumps on the groin? | | | YES | | | |  | |  | | | |  | |
|  | |  |  |  | NO | | | |  | |  | | | |  | |
|  | |  |  |  | Don’t know | | | |  | |  | | | |  | |
|  | |  |  |  | Refuse to answer | | | |  | |  | | | |  | |
| 3B720 | | Did she have any swelling or lump in the breast? | | | YES | | | |  | |  | | | |  | |
|  | |  |  |  | NO | | | |  | |  | | | |  | |
|  | |  |  |  | Don’t know | | | |  | |  | | | |  | |
|  | |  |  |  | Refuse to answer | | | |  | |  | | | |  | |
| 3B722 | | Did she have any ulcers (pits) in the breast? | | | YES | | | |  | |  | | | |  | |
|  | |  |  |  | NO | | | |  | |  | | | |  | |
|  | |  |  |  | Don’t know | | | |  | |  | | | |  | |
|  | |  |  |  | Refuse to answer | | | |  | |  | | | |  | |
| 3B724 | | Was (s)he in any way paralysed? | | | YES | | | |  | |  | | | |  | |
|  | |  |  |  | NO | | | |  | | ➡ 3B732 | | | |  | |
|  | |  |  |  | Don’t know | | | |  | | ➡ 3B732 | | | |  | |
|  | |  |  |  | Refuse to answer | | | |  | | ➡ 3B732 | | | |  | |
| 3B730 | | Did s(he) have paralysis of only one side of the body? | | | YES | | | |  | |  | | | |  | |
|  | |  |  |  | NO | | | |  | |  | | | |  | |
|  | |  |  |  | Don’t know | | | |  | |  | | | |  | |
|  | |  |  |  | Refuse to answer | | | |  | |  | | | |  | |
| 3B731 | | Which were the limbs or body parts paralysed? | | | Right side | | | |  | |  | | | |  | |
|  | |  |  |  | Left side | | | |  | |  | | | |  | |
|  | |  |  |  | Lower part of body | | | |  | |  | | | |  | |
|  | |  | | | Upper part of body | | | |  | |  | | | |  | |
|  | |  |  |  | One leg only | | | |  | |  | | | |  | |
|  | |  |  |  | One arm only | | | |  | |  | | | |  | |
|  |  |  |  |  | Whole body | | | |  | |  |  |  |  |  | |
|  |  |  |  |  | Other | | | |  | |  |  |  |  |  | |
| 3B732 | | Did (s)he have difficulty swallowing? | | | YES | | | |  | |  | | | |  | |
|  | |  |  |  | NO | | | |  | | ➡ 3B745 | | | |  | |
|  | |  |  |  | Don’t know | | | |  | | ➡ 3B745 | | | |  | |
|  | |  |  |  | Refuse to answer | | | |  | | ➡ 3B745 | | | |  | |
| 3B734 | | For how many days before death did (s)he have difficulty swallowing? | | DAYS | | | |  | |  | | |  | |  | |
| 3B740 | | Was the difficulty with swallowing with solids, liquids, or both? | | | Solids | | | |  | |  | | | |  | |
|  | |  |  |  | Liquids | | | |  | |  | | | |  | |
|  | |  |  |  | Both | | | |  | |  | | | |  | |
| 3B745 | | Did (s)he have pain upon swallowing? | | | YES | | | |  | |  | | | |  | |
|  | |  |  |  | NO | | | |  | |  | | | |  | |
|  | |  |  |  | Don’t know | | | |  | |  | | | |  | |
|  | |  |  |  | Refuse to answer | | | |  | |  | | | |  | |
| 3B750 | | Did (s)he have yellow discoloration of the eyes? | | | YES | | | |  | |  | | | |  | |
|  | |  |  |  | NO | | | |  | | ➡ 3B760 | | | |  | |
|  | |  |  |  | Don’t know | | | |  | | ➡ 3B760 | | | |  | |
|  | |  |  |  | Refuse to answer | | | |  | | ➡ 3B760 | | | |  | |
| 3B755 | | For how many days did (s)he have the yellow discoloration? | | DAYS | | | |  | |  | | |  | |  | |
| 3B760 | | Did her/his hair change in colour to a reddish or yellowish colour? | | | YES | | | |  | |  | | | |  | |
|  | |  |  |  | NO | | | |  | |  | | | |  | |
|  | |  |  |  | Don’t know | | | |  | |  | | | |  | |
|  | |  |  |  | Refuse to answer | | | |  | |  | | | |  | |
| 3B770 | | Did (s)he look pale (thinning/lack of blood) or have pale palms, eyes or nail beds? | | | YES | | | |  | |  | | | |  | |
|  | |  |  |  | NO | | | |  | |  | | | |  | |
|  | |  |  |  | Don’t know | | | |  | |  | | | |  | |
|  | |  |  |  | Refuse to answer | | | |  | |  | | | |  | |
| 3B780 | | Did (s)he have sunken eyes? | | | YES | | | |  | |  | | | |  | |
|  | |  |  |  | NO | | | |  | |  | | | |  | |
|  | |  |  |  | Don’t know | | | |  | |  | | | |  | |
|  | |  |  |  | Refuse to answer | | | |  | |  | | | |  | |
| 3B790 | | Did (s)he drink a lot more water than usual? | | | YES | | | |  | |  | | | |  | |
|  | |  |  |  | NO | | | |  | |  | | | |  | |
|  | |  | | | Don’t know | | | |  | |  | | | |  | |
|  | |  | | | Refuse to answer | | | |  | |  | | | |  | |
| **SECTION 6: HISTORY OF INJURIES/ACCIDENTS** | | | | | | | | | | | | | | |  | |

| 3E100 | Did (s)he suffer from any injury or accident that led to her/his death? | YES |  |  |  |  |
| --- | --- | --- | --- | --- | --- | --- |
|  |  | NO |  | ➡ | 3F100 |  |
|  |  |  |  |  |  |  |
|  |  | Don’t know |  | ➡ | 3F100 |  |
|  |  | Refuse to answer |  | ➡ | 3F100 |  |
| 3E102 | Was the injury intentionally inflicted by someone else? | YES |  |  |  |  |
|  |  | NO |  | ➡ | 3E113 |  |
|  |  | Don’t know |  | ➡ | 3E113 |  |
|  |  | Refuse to answer |  | ➡ | 3E113 |  |
| 3E104 | Was (s)he injured by a firearm? | YES |  |  |  |  |
|  |  | NO |  |  |  |  |
|  |  | Don’t know |  |  |  |  |
|  |  | Refuse to answer |  |  |  |  |
| 3E106 | Was (s)he stabbed, cut or pierced? | YES |  |  |  |  |
|  |  | NO |  |  |  |  |
|  |  | Don’t know |  |  |  |  |
|  |  | Refuse to answer |  |  |  |  |
| 3E108 | Was (s)he strangled? | YES |  |  |  |  |
|  |  | NO |  |  |  |  |
|  |  | Don’t know |  |  |  |  |
|  |  | Refuse to answer |  |  |  |  |
| 3E111 | Was (s)he injured by a blunt force? | YES |  |  |  |  |
|  |  | NO |  |  |  |  |
|  |  | Don’t know |  |  |  |  |
|  |  | Refuse to answer |  |  |  |  |
| 3E112 | Was (s)he injured by burns? | YES |  |  |  |  |
|  |  | NO |  |  |  |  |
|  |  | Don’t know |  |  |  |  |
|  |  | Refuse to answer |  |  |  |  |
| 3E115 | Was it a road traffic accident? | YES |  | ➡ |  |  |
|  |  | NO |  |  | 3E310 |  |
|  |  | Don’t know |  | ➡ | 3E310 |  |
|  |  | Refuse to answer |  | ➡ | 3E310 |  |
| 3E120 | What was her/his role in the road traffic accident? | Pedestrian |  |  |  |  |
|  |  | Driver or passenger in car or light vehicle |  |  |  |  |
|  |  | Driver or passenger in bus or heavy vehicle |  |  |  |  |
|  |  | Driver or passenger on a motorcycle |  |  |  |  |
|  |  | Driver or passenger on a pedal cycle |  |  |  |  |
| 3E170 | What was the counterpart that was hit during the road traffic accident? | Pedestrian |  |  |  |  |
|  |  | Stationary object |  |  |  |  |
|  |  | Car or light vehicle |  |  |  |  |
|  |  | Bus or heavy vehicle |  |  |  |  |
|  |  |  |  |  |  |  |
|  |  | Motorcycle |  |  |  |  |
|  |  | Pedal cycle |  |  |  |  |
|  |  | Other |  |  |  |  |
| 3E310 | Was (s)he injured in a fall? | YES |  |  |  |  |
|  |  | NO |  |  |  |  |
|  |  | Don’t know |  |  |  |  |
|  |  | Refuse to answer |  |  |  |  |
| 3E320 | Did (s)he die of drowning? | YES |  |  |  |  |
|  |  | NO |  |  |  |  |
|  |  | Don’t know |  |  |  |  |
|  |  | Refuse to answer |  |  |  |  |
| 3E330 | Was (s)he suffering from burns? | YES |  |  | |  |
|  |  | NO |  |  |  |  |
|  |  | Don’t know |  |  |  |  |
|  |  | Refuse to answer |  |  |  |  |
| 3E335 | Was (s)he injured by a blunt force? | YES |  |  |  |  |
|  |  | NO |  |  |  |  |
|  |  | Don’t know |  |  |  |  |
|  |  | Refuse to answer |  |  |  |  |
| 3E340 | Was (s)he injured by a plant/ animal/insect that led to her/his death? | YES |  | ➡ |  |  |
|  |  | NO |  |  | 3E500 |  |
|  |  | Don’t know |  | ➡ | 3E500 |  |
|  |  | Refuse to answer |  | ➡ | 3E500 |  |
| 3E400 | What was the plant/animal/insect? | Dog |  |  |  |  |
|  |  | Snake |  |  |  |  |
|  |  | Insect or Scorpion |  |  |  |  |
|  |  | Others |  |  |  |  |
|  |  | Don’t know |  |  |  |  |
| 3E500 | Was (s)he injured by a force of nature? | YES |  |  |  |  |
|  |  | NO |  |  |  |  |
|  |  | Don’t know |  |  |  |  |
|  |  | Refuse to answer |  |  |  |  |
| 3E510 | Was there any poisoning? | YES |  |  |  |  |
|  |  | NO |  |  |  |  |
|  |  | Don’t know |  |  |  |  |
|  |  | Refuse to answer |  |  |  |  |
| 3E520 | Was (s)he subject to violence/assault? | YES |  |  |  |  |
|  |  | NO |  |  |  |  |
|  |  | Don’t know |  |  |  |  |
|  |  | Refuse to answer |  |  |  |  |
| 3E530 | Was it electrocution? | YES |  |  |  |  |
|  |  | NO |  |  |  |  |
|  |  | Don’t know |  |  |  |  |
|  |  | Refuse to answer |  |  |  |  |

| **SECTION 7: HEALTH SERVICE UTILISATION** | | | | | | | | |  |
| --- | --- | --- | --- | --- | --- | --- | --- | --- | --- |
| 3G110 | Did (s)he receive any treatment for the illness that led to death? | | YES | |  | |  |  |  |
|  |  |  | NO | |  | | ➡ | 3H100 |  |
|  |  |  | Don’t know | |  | | ➡ | 3H100 |  |
|  |  |  | Refuse to answer | |  | | ➡ | 3H100 |  |
| 3G120 | Did (s)he receive oral rehydration salts? | | YES | |  | |  |  |  |
|  |  |  | NO | |  | |  |  |  |
|  |  |  | Don’t know | |  | |  |  |  |
|  |  |  | Refuse to answer | |  | |  |  |  |
| 3G130 | Did (s)he receive (or need) intravenous fluids (drip) treatment? | | YES | |  | |  |  |  |
|  |  |  | NO | |  | |  |  |  |
|  |  |  | Don’t know | |  | |  |  |  |
|  |  |  | Refuse to answer | |  | |  |  |  |
| 3G140 | Did (s)he receive (or need) a blood transfusion? | | YES | |  | |  |  |  |
|  |  |  | NO | |  | |  |  |  |
|  |  |  | Don’t know | |  | |  |  |  |
|  |  |  | Refuse to answer | |  | |  |  |  |
| 3G150 | Did s/he receive (or need) treatment/food through a tube passed through the nose? | | YES | |  | |  |  |  |
|  |  |  | NO | |  | |  |  |  |
|  |  |  | Don’t know | |  | |  |  |  |
|  |  |  | Refuse to answer | |  | |  |  |  |
| 3G160 | Did (s)he receive (or need) injectable antibiotics? | | YES | |  | |  |  |  |
|  |  |  | NO | |  | |  |  |  |
|  |  |  | Don’t know | |  | |  |  |  |
|  |  |  | Refuse to answer | |  | |  |  |  |
| 3G165 | Did (s)he receive (or need) antiretroviral therapy (ART)? | | YES | |  | |  |  |  |
|  |  |  | NO | |  | |  |  |  |
|  |  |  | Don’t know | |  | |  |  |  |
|  |  |  | Refuse to answer | |  | |  |  |  |
| 3G170 | Did (s)he have (or need) an operation for the illness? | | YES | |  | |  |  |  |
|  |  |  | NO | |  | | ➡ | 3G190 |  |
|  |  |  | Don’t know | |  | | ➡ | 3G190 |  |
|  |  |  | Refuse to answer | |  | | ➡ | 3G190 |  |
| 3G180 | Did (s)he have the operation within 1 month before death? | | YES | |  | |  |  |  |
|  |  |  | NO | |  | |  |  |  |
|  |  |  | Don’t know | |  | |  |  |  |
|  |  |  | Refuse to answer | |  | |  |  |  |
| 3G190 | Was (s)he discharged from hospital very ill? | | YES | |  | |  |  |  |
|  |  |  | NO | |  | |  |  |  |
|  |  |  | Don’t know | |  | |  |  |  |
|  |  |  | Refuse to answer | |  | |  |  |  |
| 3H130 | Was care sought outside the home while (s)he had this illness? | | YES | |  | | ➡ |  |  |
|  |  |  | NO | |  | |  | 3H160 |  |
|  |  |  | Don’t know | |  | | ➡ | 3H160 |  |
|  |  |  | Refuse to answer | |  | | ➡ | 3H160 |  |
| 3H140 | Where or from whom did you seek care? | | Traditional healer | |  | |  |  |  |
|  |  |  | Homeopath | |  | |  |  |  |
|  |  |  | Religious leader | |  | |  |  |  |
|  |  |  | Private hospital | |  | |  |  |  |
|  |  |  | Government hospital | |  | |  |  |  |
|  |  |  | Government health  centre or clinic |  |  | |  |  |  |
|  |  |  |  |  |  |  |  |  |  |
|  |  |  | Community-based  practitioner associated  with health system | |  | |  |  |  |
|  |  |  | Trained birth attendant | |  | |  |  |  |
|  |  |  | Private physician | |  | |  |  |  |
|  |  |  | Pharmacy | |  | |  |  |  |
| 3H150 | Record the name and address of any hospital, health centre  or clinic where care was sought  _____________________________________________________________________ | | | | | |  |  |  |
|  |  |  |  |  |  |  |  |  |  |
|  |  | |  |  |  | |  |  |  |
| 3H160 | Did a health care worker tell you the cause of death? | | YES | |  | | ➡ |  |  |
|  |  |  | NO | |  | |  | 3H180 |  |
|  |  |  | Don’t know | |  | | ➡ | 3H180 |  |
|  |  |  | Refuse to answer | |  | | ➡ | 3H180 |  |
| 3H170 | What did the health care worker say? | | |  |  | |  |  |  |
|  | _____________________________________________________________ | | |  |  | |  |  |  |
|  |  | |  |  |  | |  |  |  |
| 3H180 | Do you have any health records that belonged to the deceased? | | YES | |  | | ➡ |  |  |
|  |  |  | NO | |  | |  | 4A100 |  |
|  |  |  | Don’t know | |  | | ➡ | 4A100 |  |
|  |  |  | Refuse to answer | |  | | ➡ | 4A100 |  |
| 3H190 | Can I see the health records? | | YES | |  | | ➡ |  |  |
|  |  |  | NO | |  | |  | 4A100 |  |
|  |  |  | Don’t know | |  | | ➡ | 4A100 |  |
|  |  |  | Refuse to answer | |  | | ➡ | 4A100 |  |
| 3H200 | Record the date of the most recent (last) visit | | DAY |  |  | |  |  |  |
|  |  |  | MONTH |  |  | |  |  |  |
|  |  |  | YEAR |  |  | |  |  |  |
| 3H210 | Record the date of the last but one (second last) visit | | DAY |  |  | |  |  |  |
|  |  |  | MONTH |  |  | |  |  |  |
|  |  |  | YEAR |  |  | |  |  |  |
| 3H220 | Record the date of the last note on the health records | | DAY |  |  | |  |  |  |
|  |  |  | MONTH |  |  | |  |  |  |
|  |  |  | YEAR |  |  | |  |  |  |
| 3H230 | Record the weight (in kilograms) written at the most recent (last) visit | | [KG] | . | | |  |  |  |
|  |  |  |  |  |  |  |  |  |  |
| 3H240 | Record the weight (in kilograms) written at the last but one (second last) visit | | [KG] | . | | |  |  |  |
|  |  |  |  |  |  |  |  |  |  |
| 3H250 | Transcribe the last note on the health records | | |  |  | |  |  |  |
|  | ____________________________________________________________________ | | |  |  | |  |  |  |
|  | ____________________________________________________________________ | | |  |  | |  |  |  |
|  |  |  | |  |  | |  |  |  |
| **SECTION 8: BACKGROUND AND CONTEXT** | |  | | | |  |  |  |  |
| 4A100 | In the final days before death, did s/he travel to a hospital or health facility? | YES | | | |  | ➡ |  |  |
|  |  | NO | | | |  |  | 4A150 |  |
|  |  | Don’t know | | | |  | ➡ | 4A150 |  |
|  |  | Refuse to answer | | | |  | ➡ | 4A150 |  |
| 4A110 | Did (s)he use motorised transport to get to the hospital or health facility? | YES | | | |  |  |  |  |
|  |  | NO | | | |  |  |  |  |
|  |  | Don’t know | | | |  |  |  |  |
|  |  | Refuse to answer | | | |  |  |  |  |
| 4A120 | Were there any problems during admission to the hospital or health facility? | YES | | | |  |  |  |  |
|  |  | NO | | | |  |  |  |  |
|  |  | Don’t know | | | |  |  |  |  |
|  |  | Refuse to answer | | | |  |  |  |  |
| 4A130 | Were there any problems with the way (s)he was treated (medical treatment, procedures, interpersonal attitudes, respect, dignity) in the hospital or health facility? | YES | | | |  |  |  |  |
|  |  | NO | | | |  |  |  |  |
|  |  | Don’t know | | | |  |  |  |  |
|  |  | Refuse to answer | | | |  |  |  |  |
| 4A140 | Were there any problems getting medications, or diagnostic tests in the hospital or health facility? | YES | | | |  |  |  |  |
|  |  | NO | | | |  |  |  |  |
|  |  | Don’t know | | | |  |  |  |  |
|  |  | Refuse to answer | | | |  |  |  |  |
| 4A150 | Does it take more than 2 hours to get to the nearest hospital or health facility from the deceased's household? | YES | | | |  |  |  |  |
|  |  | NO | | | |  |  |  |  |
|  |  | Don’t know | | | |  |  |  |  |
|  |  | Refuse to answer | | | |  |  |  |  |
| 4A160 | In the final days before death, were there any doubts about whether medical care was needed? | YES | | | |  |  |  |  |
|  |  | NO | | | |  |  |  |  |
|  |  |  |  |  |  |  |  |  |  |
|  |  | Don’t know | | | |  |  |  |  |
|  |  | Refuse to answer | | | |  |  |  |  |
| 4A170 | In the final days before death, was traditional medicine used? | YES | | | |  |  |  |  |
|  |  | NO | | | |  |  |  |  |
|  |  | Don’t know | | | |  |  |  |  |
|  |  | Refuse to answer | | | |  |  |  |  |
| 4A180 | In the final days before death, did anyone use a telephone or cell phone to call for help? | YES | | | |  |  |  |  |
|  |  | NO | | | |  |  |  |  |
|  |  | Don’t know | | | |  |  |  |  |
|  |  | Refuse to answer | | | |  |  |  |  |
| 4A190 | Over the course of illness, did the total costs of care and treatment prohibit other household payments? | YES | | | |  |  |  |  |
|  |  | NO | | | |  |  |  |  |
|  |  | Don’t know | | | |  |  |  |  |
|  |  | Refuse to answer | | | |  |  |  |  |
| **SECTION 9: OPTIONAL OPEN NARRATIVE** | | | | | | | | |  |
| 5A100 | Narrative Description | | | | | | | |  |
|  |  | | | | | | | |  |
|  |  |  |  |  |  |  |  |  |  |

| **SECTION 10: DEATH CERTIFICATE** | | | | | |
| --- | --- | --- | --- | --- | --- |
| 6H260 | Was a death certificate issued? | YES |  | ➡ |  |
|  |  | NO |  |  | END |
|  |  | Don’t know |  | ➡ | END |
|  |  | Refuse to answer |  | ➡ | END |
| 6H270 | Can I see the death certificate? | YES |  | ➡ |  |
|  |  | NO |  |  | END |
|  |  | Don’t know |  | ➡ | END |
|  |  | Refuse to answer |  | ➡ | END |
| 6H280 | Record the immediate cause of death from the certificate (line 1a) *  ____________________________________________________________ | |  | Duration 1(a) | |
|  |  |  |  |  |  |
| 6H290 | Record the first antecedent cause of death from the certificate (line 1b) ____________________________________________________________ | |  | Duration 1(b) | |
|  |  |  |  |  |  |
| 6H300 | Record the second antecedent cause of death from the certificate(line 1c)  ___________________________________________________________________ | |  | Duration1(c) | |
|  |  |  |  |  |  |
| 6H310 | Record the third antecedent cause of death from the certificate (line 1d) ___________________________________________________________________ | |  | Duration 1(d) | |
|  |  |  |  |  |  |
| 6H320 | Record the contributing cause(s) of death from the certificate (part 2) _____________________________________________ | |  |  |  |
